# Development on Solid Polymer Electrolytes for Electrochemical Devices

**DOI:** 10.3390/molecules26216499

**Published:** 2021-10-28

**Authors:** Li Ping Teo, Mohd Hamdi Buraidah, Abdul Kariem Arof

**Affiliations:** Centre for Ionics University of Malaya, Physics Department, Faculty of Science, University of Malaya, Kuala Lumpur 50603, Malaysia; lpteo@um.edu.my (L.P.T.); mhburaidah@um.edu.my (M.H.B.)

**Keywords:** solid polymer electrolytes, ionic conductivity, ionic transport, plasticizers, fillers, electrochemical devices

## Abstract

Electrochemical devices, especially energy storage, have been around for many decades. Liquid electrolytes (LEs), which are known for their volatility and flammability, are mostly used in the fabrication of the devices. Dye-sensitized solar cells (DSSCs) and quantum dot sensitized solar cells (QDSSCs) are also using electrochemical reaction to operate. Following the demand for green and safer energy sources to replace fossil energy, this has raised the research interest in solid-state electrochemical devices. Solid polymer electrolytes (SPEs) are among the candidates to replace the LEs. Hence, understanding the mechanism of ions’ transport in SPEs is crucial to achieve similar, if not better, performance to that of LEs. In this paper, the development of SPE from basic construction to electrolyte optimization, which includes polymer blending and adding various types of additives, such as plasticizers and fillers, is discussed.

## 1. Introduction

In any electrochemical devices, the electrolyte is viewed as the core constituent separating the cathode and anode, and it serves as the medium for charge transport. The electrolytes in electrochemical cells exist in three forms, viz liquid, gel and solid. Liquid electrolytes (LEs) are usually found to be highly conducting; thus, they are favored in all kinds of electrochemical devices, namely lithium-ion batteries, dye-sensitized solar cells (DSSCs), quantum dot sensitized solar cells (QDSSCs), fuel cells, electrochemical double layer capacitors (EDLCs), electrochromic devices (ECDs) and so on. However, LEs are sometimes volatile and vulnerable to evaporation, leakage and corrosion in the long-term. This will lead to stability and safety issues of the electrochemical devices. Moreover, LEs can also cause the formation of solid electrolyte interphase (SEI) at the anode surface not only in Li-ion batteries but also for all alkali-metal-ion batteries. This can bring adverse effects on their performance. On the other hand, gel-type electrolytes that inherit both characteristics from electrolytes in liquid and solid forms can reduce complications due to safety, but they suffer from structural instability and low mechanical property. Hence, electrolytes in the solid state are the better and more trustworthy choice to resolve the safety problems without compromising the structural and mechanical traits.

Solid polymer electrolyte (SPE), which is fundamentally made up from dissolved conducting salt within the polymer host, was first discovered by three polymer chemists from Sheffield, UK, i.e., Fenton, Parker and Wright, about 48 years ago [1]. Then, in 1978, Armand and co-workers [2] envisioned the promising implementation of SPE in solid-state batteries. Since then, research on SPE has improved tremendously. In order for SPE to be utilized in electrochemical devices, it must have reasonably good ionic conductivity, good chemical, and thermal and mechanical stabilities, as well as good interfacial contact with electrodes. SPE is considered safe, due to the absence of volatile and flammable solvents, as well as flexible, since it can accommodate volume changes in electrodes during charging/discharging reactions of electrochemical cells (namely in Li-ion batteries). Furthermore, SPE can be prepared in various shapes and desired designs to meet the needs of electrochemical devices. It can be prepared as an ultra-thin membrane which is lightweight, thus offering promising high energy and power density. SPE is unique compared to liquid electrolyte in the sense that the polymer matrix is in charge for the solvation of ions, while the ions are located at sites which are flexible and can travel within the polymer segments. In order to be functional in electrochemical devices, the SPE must fulfil some prerequisites [3]:

- Good ambient ionic conductivity of the order between 10^−4^ and 10^−2^ S cm^−1^.
- Wide electrochemical stability window.
- Mechanically stable in order to be safe and durable for devices operation
- Thermally stable when in contact with electrode components.
- Chemical and electrochemical compatibility with electrodes.
- Obtainability—the raw materials should be abundantly found and low-priced.

A basic SPE contains a polymer and a salt. The salt is dispersed in the polymer matrix, and the ions can conduct through the polymer chains. In order for the polymer to function as host in polymer electrolytes, the macromolecules must possess polar groups which have lone-pair electrons for dative bonding with the cation from the salt. Both synthetic and natural polymers can serve as polymer matrices in SPEs as long as they have functional group(s) to fulfil the complexation criterion. Various polymer electrolytes have been developed from polymers, such as poly(ethylene oxide) (PEO) [1,4,5], cellulose [6], polyvinylidene fluoride (PVDF) [7], poly(vinyl alcohol) (PVA) [8], polyacrylonitrile (PAN) [9], chitosan [10], poly(methyl methacrylate) (PMMA) [11], starch [12] and poly(vinylidene fluoride-hexafluoropropylene) (PVDF-HFP) [13], to name a few. This review presents some achievements on both the synthetic polymers and biopolymers as polymer host in SPEs used, along with working and counter electrodes in various electrochemical devices namely lithium-ion batteries, sodium-ion batteries, DSSCs, QDSSCs, fuel cells, supercapacitors, EDLCs, ECDs, proton-conducting batteries and proton-exchange membrane fuel cells (PEMFCs). Here, we pay special attention to the conductivity aspect of SPEs in these electrochemical devices. Different types of approaches taken by researchers to improve the ionic conductivity of SPEs are also discussed. 

## 2. Ion Transport Properties and Conduction Mechanism in SPEs

Ionic conductivity is one vital characteristic in determining whether the SPE is practical and efficient for device applications. The ionic conductivity of an electrolyte is calculated by using the bulk resistance from the Nyquist impedance plot. The electrolyte is subjected to a small potential typically 10 mV to ensure a linear current–voltage relationship for impedance measurement. According to the Nernst–Einstein relationship, the ionic conductivity, *σ*, is directly proportional to the diffusion coefficient of ions, *D*, as shown in the following equation:

(1) $$\sigma =\frac{q^{2}n}{k_{b}T}D$$

where *q* is elementary charge, *n* is the number of ions, *k_b_* is Boltzmann constant and *T* is temperature. Since the electrolyte consists of salts that can ionize into cations and anions, Equation (1) can be written as follows:

(2) $$\sigma =\sigma_{+}+\sigma_{-}=\frac{q^{2}}{k_{b}T}\;\left(n_{+}D_{+}+n_{-}D_{-}\right)$$

where $\sigma_{+}$ and $\sigma_{-}$ are the ionic conductivity of cations and anions respectively, $n_{+}$ is the number of cations, $n_{-}$ is the number of anions, $D_{+}$ is the cationic diffusion coefficient and $D_{-}$ is the anionic diffusion coefficient. The movement of ions are varied in the electrolyte systems depending on the solvation environment (liquid, solid and gel). 

As aforementioned, a polymer electrolyte generally consists of inorganic salts solvated in a polymer matrix that contains functional groups, such as -OH, -C=O, -NH_2_ and -COC-. The functional groups help to solvate salts into ions via electrostatic interaction. It has been acknowledged for the past three decades that the motion of ions in polymer matrix is coupled with the segmental movement of the polymer chains, and this is described as the Brownian motion by many researchers [14]. Therefore, it is believed that the ion movements can only occur in amorphous polymers where the polymer chains are freely moveable to transport the ions. It is well-known that the motion and flexibility of polymer chains are higher in polymer matrix that possess low glass transition temperature, *T*_g_. Hence, the selection of polymer is crucial in the development of polymer electrolyte. The relation between conductivity and *T*_g_ can be explained by the Vogel-Tammann-Fulcher (VTF) equation:

(3) $$\sigma =\sigma_{o}T^{1/2}exp\left(\frac{-B}{T-\left(T_{g}-50K\right)}\right)$$

where $\sigma_{o}$ is the prefactor and *B* is related to the activation energy. *T*_g_ can also be determined from differential scanning calorimetry (DSC). Based on Equations (2) and (3), the conductivity of a polymer electrolyte can be amplified by increasing the number density of mobile ions and lowering the *T*_g_. Another approach is by decoupling ion diffusion with the polymer chains. A simplest way to fulfil the above criteria is by adding high dielectric constant plasticizers into the polymer electrolyte. A high dielectric constant can weaken the attraction forces between the ions in the salt and thus enhances the salt dissociation and the increase in number density of mobile ions [3]. Plasticizers also facilitate the pathway of ions transportation by increasing the amorphousness of the polymer electrolyte film and providing the additional coordination sites for ions to conduct.

Whu and Wick [4] have investigated the role of propylene carbonate (PC) as plasticizer in PEO-based electrolytes having lithium bis(trifluoromethanesulfonyl)imide (LiTFSI) via Monte Carlo and molecular dynamics simulations. They found that the conductivity showed significant improvement with the addition of PC due to the increase in mobility of TFSI^-^ ions [4]. The lithium ions’ diffusion remained unchanged and showed stronger interaction with the ethylene oxide oxygen as compared to the carbonate oxygen. Radial distribution function of lithium also showed weak binding between lithium and oxygen atom of TFSI^-^ in the presence of PC [4]. Therefore, the probability of lithium ions’ transportation through the oxygen atom of PEO was higher than that of carbonate and TFSI^-^ oxygen [4].

Webb and co-workers [5] calculated the diffusion of lithium ions in different types of polymers, namely PEO, poly(methylene oxide) (PMO), poly(propylene oxide) (PPO), poly(trimethylene oxide) (PTMO), poly(ethylene oxide-alt-methylene oxide) (P(EO-MO)) and poly(ethylene oxide-alt-trimethylene oxide) (P(EO-TMO), using molecular dynamics and dynamic bond percolation models. They have assigned short molecular dynamics trajectories to identify the distribution of the solvation sites and used it in dynamic bond percolation model to calculate the ionic hopping rate. According to them, the largest number of site density belongs to the polymer with the highest oxygen atom content which is PMO (oxygen to carbon atomic ratio is 1:1). This is followed by P(EO-MO) (2:3), PEO (1:2), P(EO-TMO) (2:5), PPO (1:3) and PTMO (1:3). Even though the ratio of oxygen to carbon atoms for PPO and PTMO are the same, the site density is slightly higher in PPO which may be due to the arrangement of atoms in the PPO [5]. The rate of hopping sites, $k_{0},$ with distance, *r*, can be calculated by using the following equation:

(4) $$k_{0}\left(r\right)=\tau^{-1}e^{-E_{dis}\left(r\right)/k_{b}T}\;e^{-E_{\lambda }\left(r\right)/k_{b}T}$$

where $\tau^{-1}$ is frequency, and $E_{dis}$ and $E_{\lambda }$ are the dissociation and reorganization energies, respectively. Based on the calculation, the authors found that PMO exhibited the smallest value of $k_{0},$ whereas that of PPO was the largest [5]. They have used these values to calculate lithium-ion diffusion, using the kinetic Monte Carlo simulation. Among the polyethers studied, the diffusion of lithium ions in P(EO-TMO) was the highest [5]. 

Diffusion coefficient of ions, *D*, can also be determined from the following equation:

(5) $$D=\frac{d^{2}}{4\tau_{2}\delta^{2}}$$

where *d* is the thickness of the electrolyte, $\tau_{2}=1/\omega$ and $\delta =dk^{-1}/A\varepsilon \varepsilon_{0}$ (where *ω* is the angular frequency taken at the minimum value of impedance in Bode plot; $k^{-1}$ is double layer capacitance; *ε* is dielectric constant of the electrolyte; and $\varepsilon_{0}$ is the permittivity in free space). Based on Equation (5), *D* in PVA-based polymer electrolyte containing potassium iodide salt has been calculated [6]. The $k^{-1}$ value was obtained by fitting the Nyquist plot of the electrolyte. Since the complex impedance plot shows only a tilted spike, the plot has been fitted based on the series connection of bulk resistance, *R_b_*, and constant phase element, CPE. 

The total impedance (real, $Z^{\prime }$ and imaginary, $Z^{\prime \prime }$) can be described as follows:

(6) $$Z^{\prime }=R_{b}+cos\;\left(\pi p/2\right)/k^{-1}\omega^{p}$$

(7) $$Z^{\prime \prime }=sin\;\left(\pi p/2\right)/k^{-1}\omega^{p}$$

where *p* is a fraction of a right angle. The mobility, *μ*, and number density, *n*, of ions can be obtained by using the following equations:

(8) $$\mu =qD/k_{b}T$$

(9) n=σ/qμ

It is evident that Equation (1) is the combination of Equations (8) and (9). The calculated transport properties (*D*, *n* and *μ*) have been used to explain the conductivity behavior in PVA-based electrolyte containing different salt concentrations [8]. Relationship between *σ*, *n* and *μ* can be expressed as follows:

(10) σ=qnμ

In our earlier studies [8], it was found that the ionic mobility has contributed more in the highest conducting electrolyte, since *n* decreased due to the establishment of ion pairs and ionic aggregates. Noor has calculated *n* for lithium triflate in gellan-gum-based SPE by using the method from Fourier transform infrared (FTIR) spectroscopy [9,15]. Areas of free ions (*A_f_*), ion pairs (*A_p_*) and ion aggregates (*A_a_*) have been identified via FTIR deconvolution in wavenumber region between 1030 and 1060 cm^−1^, and their percentage can be calculated based on the following equations:

(11) $$\mathrm{Free}\;\mathrm{ions}\;(\%)=\frac{A_{f}}{A_{f}+A_{p}+A_{a}}\times 100$$

(12) $$\mathrm{Ion}\;\mathrm{pairs}\;(\%)=\frac{A_{p}}{A_{f}+A_{p}+A_{a}}\times 100$$

(13) $$\mathrm{Ion}\;\mathrm{aggregates}\;(\%)=\frac{A_{a}}{A_{f}+A_{p}+A_{a}}\times 100$$

From Equation (11), *n* can be calculated from the following:

(14) $$n=\frac{M\times N_{A}}{V}\times 2\times percentage\;of\;free\;ions$$

where *N_A_* is the Avogadro’s number, *M* is number of moles of lithium triflate and *V* is the electrolyte’s volume. 

The *n* value can also be calculated from the famous Rice and Roth model which was originally developed to describe the conductivity behavior for superionic conductors [16]. The equation is as follows:

(15) $$\sigma =\frac{2}{3}\left[\frac{{\left(Zq\right)}^{2}}{mk_{b}T}\right]nE_{A}\tau \;exp\left[-\frac{E_{A}}{k_{b}T}\right]$$

where *E_A_* is the activation energy; *q* is the electron charge; *m* is the mass of ions; *Z* is the vacancy of conducting species; and τ=r/ν, where *r* is the distance between two coordinating sites and ν is the velocity of ions, which is given as follows:

(16) $$\nu =\sqrt{\frac{2E_{A}}{m}}$$

*E_A_* is the minimum energy required for an ion to hop in between sites, and it can be obtained from the Arrhenius equation, as shown below:

(17) $$\sigma =\sigma_{0}exp\left(\frac{E_{A}}{k_{b}T}\right)$$

where $\sigma_{0}$ is the pre-exponential factor. A low *E_A_* value is required for high conductivity. In general, the mechanism of ion transport in polymer electrolytes is via hopping and coupled with the segmental motion of the polymer [3]. However, Grotthuss motion which is another type of hopping mechanism has been proposed to describe proton conduction in chitosan-based SPE containing ammonium iodide (NH_4_I) [10]. Chitosan structure is rigid and possesses high *T_g_*, thus resulting in minimal chain motion. This led to the proposal of the Grotthuss model as the proton-conduction mechanism in the SPEs. Grotthuss mechanism is the proton conduction process where the proton hops between the coordination sites decoupled from the polymer chain motion. This mechanism is also known as proton hopping. Besides these factors, other parameters that can influence the feasibility of polymer electrolytes in applications for electrochemical cells include ion (cationic and anionic) transference number (*t_ion_*), degree of crystallinity, etc. 

## 3. Basic Solid Polymer Electrolytes 

A simple SPE consists of a polymer and a salt where both are dissolved in a solvent before the solvent is allowed to evaporate, while the ions continue to be free and mobile inside the polymer matrix [3]. The salt can be either inorganic or organic salt but when used in DSSCs application, the SPE must contain additional materials, i.e., iodine (I_2_) crystals to act as redox mediator, while the salt must be iodide-based salts, since iodide/triiodide (I^−^/I_3_^−^) redox couple is commonly used [17]. Likewise, for application in QDSSCs, the basic SPE should have sulfide/polysulfide (S^2−^/S_x_^2−^) redox mediator with sulfide-based salts. Similarly, the salt should be lithium-based, sodium-based, magnesium-based and proton-based if it is going to be employed in cells of Li-ion, Na-ion and Mg-ion and proton batteries as well as PEMFCs. Besides this, the salt must meet the criterion of having low lattice energy for easy solvation in polymer matrix [3]. Table 1 and Table 2 list some of the most popular polymers and salts used in polymer electrolytes, along with their special traits that enable them to be favored among others by the researchers worldwide. The *T_g_* of polymer will affect the crystallinity of the electrolytes, and usually low *T_g_* is preferred. Meanwhile, the lattice energy of salt will determine how easy it can dissociate in the polymer matrix. The lattice energies of the salts in Table 2 are calculated in this work, using the Kapustinskii equation [18]. All materials have both their own benefits and drawbacks. For instance, PEO, being the first polymer host used in polymer electrolytes and still remaining one of the most popular polymers until now, is favored for its talent to solvate many types of salts and exhibit good mechanical strength and thermal stability. It is a semicrystalline macromolecule, and thus, its crystalline part restricts the increment of conductivity. As for the salts, lithium perchlorate (LiClO_4_), as an example, shows good ionic conductivity, but it is a strong oxidizer, hence making it explosive, and so it must be handled with caution. As for the solvent choice, certain features, including dielectric constant, melting point, boiling point and viscosity, have to be taken into consideration for salt dissociation and solvation of polymer. Some frequently employed solvents are summarized in Table 3, along with their physical attributes. 

### 3.1. Preparation Methods of SPEs

SPEs can be prepared via several methods, including solution casting [31], phase inversion [32], photopolymerization [33] and electrospinning [31] techniques, to name a few. Solution cast or solvent casting method is the most conventional and easy way to prepare electrolyte film. In this procedure, the polymer and salt are dissolved in an appropriate solvent before casting onto suitable substrates (e.g., glass plates). The solvent is then evaporated after being left to dry either at ambient condition or elevated temperature inside oven. Solution casting route can be performed at room temperature atmosphere or inside glove box, depending on its needs. After complete drying, the SPE is a free standing membrane with thickness usually in millimeter range. Solangi and co-authors [31] have compared PVA-based SPE containing potassium iodide (KI), potassium chloride (KCl) and sodium chloride (NaCl) salts prepared by using electrospinning and solution-casting techniques. Regardless of which salt was used, the electrolyte fabricated via the former method demonstrated superior ionic conductivity (5.95 × 10^−6^ S cm^−1^ for NaCl-containing SPE) than that prepared by the latter (1.87 × 10^−6^ S cm^−1^ for SPE having NaCl) [31]. The electrospinning approach can produce film of fibers morphology with a large surface-area-to-volume ratio. Moreover, the electrospun membranes were more thermally stable than the solution-cast prepared films. Among the three salts, electrolytes comprising NaCl exhibited the highest conductivity irrespective of the preparation method [31]. Nonetheless, there are many parameters that need to be controlled and optimized in electrospinning process such as polymer molecular weight and solution viscosity, conductivity and surface tension. Furthermore, the electrospinning setup conditions, e.g., flow rate, applied voltage and distance from tip of syringe containing solution to collector, are tedious and time-consuming. In contrast to electrospinning, the solution-casting route is a more straightforward and convenient approach for membrane preparation. 

The phase inversion technique can produce porous structure which is beneficial for ionic transport. Using this method, SPE membrane consisting of PDVF-HFP, LiClO_4_ and cerium oxide (CeO_2_) exhibited sponge-like morphology with tiny pores and ionic conductivity of 2.50 × 10^−3^ S cm^−1^ at 25 °C [32]. In this procedure, a solution containing PVDF-HFP, CeO_2_ and NMP solvent was stirred for 24 h before being coated onto a glass plate via the doctor-blade method. This was followed by immersion in deionized water (about 3 to 5 h) for solvent extraction and phase inversion, which took place before drying under vacuum-pressure condition. The last step was to soak the membrane in LiClO_4_ liquid electrolyte for 6 h [32]. Liu et al. [34] have combined phase-inversion and chemical-reaction routes to obtain PVDF-PAN-SiO_2_-LiPF_6_ SPE membrane that gave ambient conductivity of 3.32 × 10^−3^ S cm^−1^. For comparison, the same composition membrane has been prepared by phase-inversion method, and it was found that the ambient conductivity was 2.83 × 10^−3^ S cm^−1^ [34]. The higher conductivity obtained by the membrane prepared by the combined method was attributed to larger porosity and better homogeneous pores distribution as compared to that developed from the phase-inversion technique only [34]. The former also showed better properties in terms of heat resistance, mechanical strength and electrochemical stability than the latter [34]. An unconventional way to prepare SPE films is through dry-mixing without the use of solvent. Dry-mixing is combined with hot-pressing. Eriksson et al. [35] have obtained thin films of SPEs containing poly(3,3-dimethylpentane-2,4-dione) as the polymer matrix and LiTFSI salt after pressing the dried homogenous mixture between two polytetrafluoroethylene (PTFE) plates, under high pressure (2 MPa) and temperature (100 °C) conditions, inside an argon-filled glove box for 1 h. The polymer was desiccated in vacuum state before uniformly mixing and pulverizing the polymer and salt. Although the ambient conductivity value was low, of the order of 10^−8^ S cm^−1^, it gave a rather high Li^+^ ion transference number (*t_Li_*_+_ = 0.70) at 80 °C [35]. For the sake of comparison, the LiTFSI-containing SPE but with PEO as polymer host has been prepared by the same method and it showed a higher ambient conductivity (10^−5^ S cm^−1^) with low Li^+^ transference number (*t_Li_*_+_ = ~0.15) [35]. High cationic transference number is useful for application in Li-ion batteries. Table 4 shows some selected works of simple SPEs systems available in the literature. 

### 3.2. Strategies to Improve Ionic Conductivity of SPEs

In order to boost the ionic conductivity of SPEs, there are many approaches which have been taken by researchers, as stated in the following subsections.

#### 3.2.1. Incorporation of More Than One Salt

One viable approach to boost the ionic conductivity of SPEs is to add another salt. The combination of two or more salts in the right proportions can give synergistic outcomes. In most cases, the sum concentration of salts is the same as the highest conducting electrolyte that contained only on one type of salt. This means that the total weight of salts remained unchanged, but an appropriate amount for salt one has been substituted by salt two. Since the amount of each salt in a mixed salt electrolytes is lower than the concentration of salt in single-salt electrolyte systems, the probability of ion recombination to form ion pairs will be reduced in former case, and thus, there are more free ions for conduction [48,49]. In addition, decrement in *T_g_* and degree of crystallinity (%χ_c_) of the electrolyte are observed in mixed salt systems [48]. Tao and Fujinami [50] reported that highest conductivity was achieved at 5 × 10^−5^ S cm^−1^ (30 °C) in PEO-based SPE containing lithium borate and lithium aluminate salts as compared to PEO electrolyte with one salt only regardless which one. Only minimal capacity fading was observed in LiNi_0.8_Co_0.2_O_2_/Li cell with double salt-containing SPE after 30th cycle, but no results were given for that of single salt [50]. The type of cation and anion determine the electrochemical performance of devices. For Li-ion, Na-ion and Mg-ion cells, it is obvious that dissimilar anions influence the performance of the devices. For DSSCs, the variation of cations since the anion is fixed following the redox mediator used. Studies of mixed salt systems containing different cations [51,52] or dissimilar anions [49,53,54] have shown superior ionic conductivity than single salt systems. For instance, higher conductivity was obtained for an electrolyte based on PEO-CaBr_2_-CaI_2_ (30:1:1) than for a PEO-based electrolyte with only one salt, i.e., either calcium iodide (CaI_2_) or calcium bromide (CaBr_2_) [52]. Similarly, enhanced conductivity has been detected for PEO electrolyte having dual salts of zinc bromide (ZnBr_2_) and lithium bromide (LiBr) [51] and PEO-based electrolyte comprising the same compositions of binary salts of copper trifluoromethanesulfonate (Cu(CF_3_SO_3_)_2_) and zinc trifluoromethanesulfonate (Zn(CF_3_SO_3_)_2_) [55]. 

The incorporation of double salts does not always warrant higher conductivity than single salt but can still result in better performance of electrochemical devices, since conductivity is not the sole influencing factor. This has been showcased in the work of Dissanayake et al. [56], who have prepared PEO-based SPE with KI and TPAI salts via the solution-cast method. SPE containing sole salt of KI gave higher conductivity than electrolyte having double salts. Conductivity was lowest for electrolyte with lone TPAI salt since its cation is larger than K^+^ and thus less mobile. Moreover, the degree of crystallinity for PEO-TPAI electrolyte was higher than that of PEO-TPAI-KI SPE [56]. DSSC employing hybrid salts displayed the best performance, with an efficiency (*η*) of 4.22% and short circuit current density (*J_sc_*) of 8.00 mA cm^−2^, followed by cells having single TPAI (*J_sc_* = 6.38 mA cm^−2^; *η* = 3.54%) and KI (*J_sc_* = 6.52 mA cm^−2^; *η* = 3.09%) under illumination of 100 mW cm^−2^. Their open-circuit voltage (OCV) values were 0.69, 0.77 and 0.75 V for KI, TPAI and KI+TPAI electrolytes, respectively [56]. A large-sized cation boosted OCV value, while it reduced *J_sc_*, as can be seen when comparing the two single salt-containing electrolytes. The addition of a second salt decreased the cation conduction and enhanced *J_sc_*, leading to a higher *η* [56]. 

Zhao and co-workers [57] have studied the influence of primary, secondary and ternary salts on PEO-based SPEs incorporated with halloysite nanoclay (HNC) for application in Li-ion batteries. For PEO-LiTFSI-HNC SPEs, the ambient conductivity was ~5.62 × 10^−5^ S cm^−1^, and there was a slight increment when a secondary salt, i.e., lithium bis(oxalate)borate (LiBOB), was added, but then there was a decrement in conductivity (~1.99 × 10^−5^ S cm^−1^) was observed with the addition of a third salt, lithium nitrate (LiNO_3_) [57]. Nonetheless, *T_g_* values were enhanced from −47.02 to −40.48 °C for single to ternary salt-containing SPEs [57]. Mechanical strength also improved as proven from dynamic mechanical analysis (DMA). The authors have concluded that each respective salts have their own role with LiTFSI being the main contributor to ionic conduction, whereas the presence of LiBOB and LiNO_3_ salts was to ensure the emergence of cathode electrolyte interphase and solid electrolyte interphase SEI on cathode and anode, respectively [57]. Each of these salts have complemented each other and yielded Li-ion-cell performance with better electrochemical stability. Cells employed one and two salts suffered failure within the first five cycles. In contrast, the lithium nickel cobalt manganese oxide (NMC)/Li cell with SPE containing trio salts exhibited the discharge capacity of ~116 mAh g^−1^ at 60th cycle [57]

#### 3.2.2. Modification on Polymer Host

The partial crystallinity nature of polymer host is usually the main culprit behind the low conductivity of SPEs. This issue can be resolved by making some alteration on the polymer itself. For example, biopolymers, i.e., cellulose and chitin, are highly crystalline with rigid polymer chain backbone, even though they can still serve as host in polymer electrolyte. Cellulose can be chemically modified into methyl cellulose (MC), carboxymethyl cellulose (CMC), hydroxypropyl cellulose (HPC) and sodium carboxymethyl cellulose (NaCMC), to name a few. Similarly, chitosan structure can be altered into carboxymethyl chitosan, hexanoyl chitosan (HCh), N-methylene phosphonic chitosan, chitosan–N-propyl sulfonic acid, etc. Besides reducing crystallinity, the modified polymer will have greater solubility to dissolve in various solvents, as compared to its predecessor. N-phthaloyl chitosan (PhCh) is the product obtained after the chemical adjustment of chitosan in a solution mixture of phthalic anhydride and DMF under a nitrogen gas environment. Chitosan has been successfully transformed into PhCh with the presence of peaks belonging to the pthalimido functional group at wavenumbers 1713 and 1733 cm^−1^, as proven by FTIR analysis [58]. Dilute acids of formic, acetic, butyric, lactic, maleic and malic are some solvating agents for chitosan [59,60], but the water content may deteriorate the performance of some electrochemical devices, such as Li-ion batteries and DSSC. PhCh can dissolve in pyridine, DMAc, DMF, DMSO and *m*-cresol [61,62]. Aziz et al. [58] reported that PhCh is more amorphous and thermally stable than the antecedent, chitosan. The former requires a higher decomposition temperature (356 °C) than the latter (290 °C). PhCh-based SPE consisting of NH_4_SCN prepared via solution-casting method displayed conductivity of 2.42 × 10^−5^ S cm^−1^ at 25 °C and *t_ion_* = 0.91 [58]. The PhCh film had the ambient conductivity of ~1.93 × 10^−10^ S cm^−1^ [58]. O-nitrochitosan is another derivative of chitosan after reacting with sodium hydroxide and fumed nitric acid, as synthesized by Rahman et al. [63]. The appearance of new bands at wavenumbers 1355 and 1646 cm^−1^ were attributed to nitro (-O-N=O) functional groups, as evidenced by the FTIR spectrum of o-nitrochitosan [63]. The ambient conductivity was greatly enhanced for the modified chitosan film (5.22 × 10^−6^ S cm^−1^) at room temperature, while the unmodified chitosan film exhibited low conductivity of 8.88 × 10^−10^ S cm^−1^ [63]. The large increment was attributed to the high electronegativity of nitro group [63]. Although no dopant salt was incorporated, the authors believed that o-nitrochitosan can be a promising polymer electrolyte membrane in PEMFC [63]. 

PEO is excellent to solvate Li^+^ ion, which is especially useful for application in Li-ion cells, even though its crystalline portion hampers the conductivity enhancement. Usually, the conductivity of PEO electrolyte containing lithium salts is no greater than 10^−4^ S cm^−1^ under ambient condition with a low value of lithium-ion transference number (*t_Li_*_+_ < 0.40) [64]. Luckily, its chemical structure can be modified and incorporated with various types of functional groups or moieties. Another strategy to improve the conduction of PEO-based electrolyte is to modify its linear chain into hyper-branched structure. Jing et al. [64] have prepared LiTFSI-containing SPE based on PEO having a hyper-branched arrangement and grafting with another PEO of linear chains to improve the ionic conductivity without sacrificing its mechanical strength. An increment by one order of magnitude was observed when compared to SPE having PEO linear chain, which was attributed to reduction in crystallinity [64]. 

Besides polymer modification, the polymer can also undergo copolymerization, grafting, crosslinking and blending with a second polymer in order to lower the crystallization. Imperiyka and co-authors [65] have photo-copolymerized polyglycidyl methacrylate (P(GMA)) with PMMA as the polymer host. SPE with configuration P(GMA-co-MMA)-LiClO_4_ displayed the ambient conductivity of 8.70 × 10^−6^ S cm^−1^ [65]. The bonding of one polymer chain with that of another polymer is known as a crosslinking reaction. This process can take place physically and chemically (photo-induced polymerization, curing, etc.). In 2016, Lehmann et al. [66] prepared SPE containing PEO (M_w_ 2000 g mol^−1^) crosslinked with poly(ethyleneimine) (PEI) (M_w_ 600 g mol^−1^) and LiTFSI salt and obtained the conductivity of 3.90 × 10^−5^ S cm^−1^ at 40 °C. The values of *T_g_*, percentage of crystallinity, tensile strength and cationic transference number were −33 °C, 20%, 0.47 MPa and 0.76 (40 °C), respectively. Meanwhile, SPE, which comprises 600 M_w_ PEI, with the same salt, attained the conductivity of 1.50 × 10^−6^ S cm^−1^ at 40 °C and *T_g_* of −65 °C [66]. In 2016, Youcef et al. [67] systematically studied the concentration effect of crosslinking agent (divinylbenzene (DVB)) on SPE based on PEO crosslinked poly(ethylene glycol) diacrylate (PEGDA) with LiTFSI salt that had been prepared via polymerization under ultraviolet (UV) irradiation, using a photo-initiator, -hydroxy-2-methylpropiophenone and acetonitrile solvent. At optimum concentration of 10%mol DVB, conductivity of 1.40 × 10^−4^ S cm^−1^, lithium-ion transference number (*t_Li_*_+_) of 0.21 and high electrochemical window of 4.30 V were obtained at 70 °C [67]. At the same temperature and C/10 rate, LiFePO_4_/Li battery employing this electrolyte delivered the first discharge capacity of 138 mAh g^−1^ which was sustainable even after the 20th cycle (138 mAh g^−1^) [67]. The SPE has also been tested in Li-sulfur battery under similar conditions and acquired the capacity of 175 mAh g^−1^ after the 50th cycle, with an initial capacity of 375 mAh g^−1^ [67]. After cycling, there was no lithium dendrite formation, as evidenced through SEM micrograph. In addition to inhibit crystallinity percentage, the crosslinked electrolyte is said to have the ability to hamper lithium dendrite growth when being used in Li-ion cells and to improve dimensional stability at elevated temperatures [66,67,68,69]. 

In a separate report, PEO underwent crosslinking reaction with tetraethylene glycol dimethyl ether (TEGDME) employing 4-methyl benzophenone (MBP) as crosslinker agent, LiTFSI salt under UV radiation without solvent usage [68]. It is said that MBP can remove H^+^ from its methylene group via hydrogen abstraction to form free-radical chains [68]. Likewise, TEGDME that contained the methylene group produced free radicals. These free-radical chains have interlinked with -EO- unit of PEO chain under UV exposure to form SPE film that displayed features, such as being highly amorphous and having a uniform morphology, high mechanical strength, good flexibility, low *T_g_* of −34 °C and compatibility with Li metal electrode. At 25 °C, the SPE showed a conductivity of 0.11 × 10^−3^ S cm^−1^, *t_Li_*_+_ of 0.55 and diffusion coefficient of Li^+^ ion of 5.6 × 10^−6^ cm^2^ s^−1^ [68]. The TiO_2_/Li cell having such an electrolyte had good cyclability over 100 cycles, with a capacity of ~138 mAh g^−1^ and almost 90% capacity retention [68]. Lim et al. [70] employed an in situ polymerization procedure to prepare SPE based on crosslinking poly(ethylene glycol) diglycidyl ether (PEGDE) and bisphenol A diglycidyl ether (BADGE) with 1-ethyl-3-methylimidazolium bis(trifluoromethylsulfonyl)imide (EMIMTFSI), using PEI as crosslinker without solvent. The SPE was prepared directly on activated carbon electrode for supercapacitor application before the curing process. Such an SPE film showed a conductivity of 2.41 × 10^−3^ S cm^−1^ at 25 °C and capacitance value of 19.8 F g^−1^ at 2 mV s^−1^ scan rate with decent cyclability upon 10,000 cycles [70].

Chitosan in 1% acetic acid solution was subjected to a crosslinking process with methanesulfonic acid (MSA) and dodecylbenzene sulfonic acid sodium salt (DBSA-Na), using sulfuric acid as an agent to form chitosan-MSA and chitosan-DBSA membranes, respectively [71]. Such an action improved the proton conductivity and the thermal and mechanical stabilities of the chitosan-based membranes, which can be applied as solid electrolyte membranes in PEMFCs. At the elevated temperature of 90 °C, the proton conductivity was 3.09 × 10^−4^ S cm^−1^ for chitosan-DBSA film, whereas 2.18 × 10^−4^ S cm^−1^ was the conductivity exhibited by the chitosan-MSA membrane [71]. The non-crosslinked chitosan membrane was also prepared for comparison and showed lowest conductivity of 1.04 × 10^−4^ S cm^−1^ at same temperature [71]. The grafted copolymers consisting of natural rubber and methyl methacrylate, namely 49% PMMA-grafted NR (MG49) [48,72,73] and 30% PMMA-grafted NR (MG30) [74], have been used as polymer hosts to produce polymer electrolytes. Su’ait et al. [72] reported a conductivity of 1.00 × 10^–12^ S cm^–1^ for pure MG49 at 30 °C, and this value increased to 2.30 × 10^–7^ S cm^–1^ when MG49 was incorporated with 20 wt.% LiBF_4_ salt. Such an electrolyte of MG49-LiBF_4_ was then added to another salt (LiI and LiCF_3_SO_3_) by the same group of researchers for the mixed-salt effect [48]. It was found that the ambient conductivity increased with different salts and combination of salts as follows: LiCF_3_SO_3_ (2.15 × 10^−11^ S cm^−1^) < LiI (4.73 × 10^−9^ S cm^−1^) < LiCF_3_SO_3_+LiBF_4_ (7.71 × 10^−9^ S cm^−1^) < LiBF_4_ (1.42 × 10^−8^ S cm^−1^) < LiBF_4_+LiI (1.89 × 10^−6^ S cm^−1^) [48]. This clearly demonstrates that the ionic conductivity was enhanced significantly for the MG49-based SPE having double salts of LiBF_4_ and LiI at a ratio of 30:70, while the addition of LiCF_3_SO_3_ into the MG49-LiBF_4_ electrolyte had an adverse effect on the conductivity. The transference number of ions for the five samples was observed to increase in the same order of conductivity [48]. It can be understood that the degree of crystallinity increased in the reverse order [48]. Modified natural rubber has superior elastic features for better interfacial contact between electrolyte and electrode when applied in electrochemical devices. Another strategy to improve the properties of electrolyte is to bind the anion directly to the polymer chain to form a single-ion-conducting polymer electrolyte. Such an electrolyte consisting of poly[lithium 1-[3-(methacryloyloxy) propylsulfonyl]-1-(trifluoromethylsulfonyl) imide] (PLiMTFSI) attached to PEG-crosslinked poly(2-hydroxyethylacrylate) (PHEA) showed improved mechanical strength as compared to an electrolyte of crosslinked PEG-PHEA doped with LiTFSI, even though its conductivity is lower than the latter [75]. Nonetheless, the single-ion-conducting polymer electrolyte could minimize lithium dendrite formation and gave better results in Li-ion cells. 

#### 3.2.3. Treatment of SPEs

The polymer electrolytes can also be subjected to radiation in order to tune their properties in terms of structural, thermal, electrical and mechanical aspects. Various radiation sources, including gamma rays, X-rays, ultraviolet rays, electron, neutron and ion beams, can be employed. Solution-cast-prepared SPE based on PEO with lithium sulfate (Li_2_SO_4_) salt has been irradiated with low-energy electron beams of 8 MeV by Raghu et al. [76]. At 30 °C, the conductivity was enhanced to the order of 10^−4^ S cm^−1^ for PEO-Li_2_SO_4_ electrolyte after exposure to 30 kGy dose of electron beam from 10^−5^ S cm^−1^ for non-irradiated electrolyte. Analyses from DSC and X-ray diffraction (XRD) demonstrated that the former have a lower melting temperature (67.6 °C) and are more amorphous in nature, with reduced crystallinity, than the latter (69.4 °C melting temperature and more crystalline), while FTIR results indicated that the polymer chain was disrupted and degraded after irradiation [76]. Irradiating of Li^3+^ ion at high energy of 60 MeV on two different electrolytes of PEO-NaI and PVP-NaI showed increment in conductivity as compared to unirradiated electrolyte films [77]. Besides conductivity, higher values for number of mobile ions, ionic mobility and dielectric constant have been reported for the solution-cast-prepared SPE after ion-beam irradiation [77]. PAN-based SPE having LiBOB salt also exhibited enhancement in conductivity and transport properties (*n*, *μ* and *D*) after being radiated with a gamma-ray dose of 15 kGy [78]. Ambient conductivity was increased by 58.6 times from 1.74 × 10^−6^ S cm^−1^ to 1.02 × 10^−4^ S cm^−1^, while improvement of 12.2, 4.8 and ~4.7 times was obtained in *n*, *μ* and *D*, respectively [78]. Likewise, PVDF-LiBOB SPE has attained higher values of conductivity and ionic transport upon gamma-ray radiation as compared to non-irradiated electrolyte [79]. Hema et al. [80] also reported similar observation in PVA-PVDF-LiCF_3_SO_3_-SiO_2_ SPEs, where conductivity was boosted to 9.40 × 10^−4^ S cm^−1^ at 30 °C after gamma-ray exposure (15 Gy dose). The conductivity of such an electrolyte without radiation treatment was 1.70 × 10^−4^ S cm^−1^. Ionic transference number for the irradiated SPE also showed increment (*t_ion_* = 0.99) as compared to that of non-gamma ray treated SPE (*t_ion_* = 0.98) [80]. On the other hand, lower values for the percentage of crystallinity, *T_g_* and melting point were detected in the former than in the latter [80]. In general, it is to be noted that conductivity enhancement has been observed at optimized dosage of radiation irrespective of the sources due to polymer chain scission, whereas too much radiation dose will be detrimental to the properties of SPE, and this is attributed to crosslinking and increase in crystallinity. 

In another work of Raghu et al. [81], a comparison was made between gamma-ray (cobalt-60 source) and electron-beam (energy of 8 MeV) treatments on PEO-based SPEs of cadmium chloride (CdCl_2_) salt. Non-irradiated electrolyte exhibited the lowest conductivity value at 8.63 × 10^−6^ S cm^−1^ at 30 °C, as compared to gamma-ray-treated (0.115 S cm^−1^) and electron-beam-irradiated (0.175 S cm^−1^) electrolytes. Micrographs from scanning electron microscopy (SEM) showed less PEO spherulites in both irradiated SPEs as compared to that of non-irradiated SPE, indicating that a more amorphous region existed in the former case. In addition, the SEM image for electrolyte after electron-beam exposure displayed a homogenous morphology, while the formation of aggregates was seen in the micrograph of SPE irradiated with gamma radiation [81]. The SPEs were irradiated with electron beam and gamma ray separately at two different doses (50 and 150 kGy), and it was found that, at the 150 kGy dosage, both types of radiation gave a higher conductivity (of the order of 10^−4^ S cm^−1^) than that irradiated at the 50 kGy dose (around 10^−5^ S cm^−1^) [81]. The authors have concluded that the electron-beam treatment is an effective approach as compared to gamma-ray radiation in making the SPE more conducting due to reduction in crystallinity. Recent work illustrated contradictory results, wherein no effect was observed on the conductivity upon neutron irradiation at varied dosages (15 to 47 kGy) on PVDF–LiBOB SPEs prepared via solution-casting route [82]. Ambient conductivity was the highest at 1.27 × 10^−5^ S cm^−1^ for the non-irradiated SPE as compared to similar electrolyte after exposed to neutron radiation with lowest value at 4.30 × 10^−7^ S cm^−1^ for SPE subjected to 47 kGy dose [82]. This was attributed to decrement in *μ* and *D*, as well as higher degree of crystallinity. The authors have deduced that an improvement in mechanical integrity in the electrolyte has occurred after being irradiated with a neutron [82]. Sinha et al. [83] have observed that there was a slight decrement in conductivity at ambient temperature for the solution-cast-prepared SPE based on PEO with ammonium perchlorate (NH_4_ClO_4_) salt after being irradiated with gamma rays, followed by minor conductivity increment at 50 °C. In the following sections, additional ingredients such as secondary polymer, plasticizers, fillers and other additives that have been integrated into various SPEs are discussed. 

#### 3.2.4. Blended Solid Polymer Electrolytes

Polymer blending can be regarded as the most convenient and simplest way to prepare SPEs that are highly conducting, flexible and thermally stable and that have good mechanical strength without additional employment of materials such as initiator, linker agent, radiation sources, etc., thus indirectly making this approach more cost-effective as compared to other techniques aforementioned. In this technique, two or more polymers which are miscible in the appropriate solvent form a homogenous solution before addition of the conducting salt. The blending of polymer approach is able to (i) reduce the glass transition temperature, (ii) decrease the degree of crystallinity, (iii) increase the ionic conductivity, (iv) improve the thermal stability, (v) enhance the mechanical strength and (vi) boost the performance of electrochemical devices. There are many studies on blended SPEs available in the literature. Table 5 lists some of the systems under this category. 

Sengwa et al. [100] have investigated the effect of SPE preparation method on conductivity. The SPE comprising PEO blended with PMMA at a ratio of 1:1 and LiCF_3_SO_3_ salt has been prepared in four ways: (i) conventional solution cast coupled with hot melt-pressing; (ii) ultrasonication followed by solution casting and hot melt press; (iii) irradiation of microwave before solution-cast and hot-melt-press techniques; and (iv) ultrasonication followed by microwave exposure, solution casting and hot melt press. The authors have observed that conductivity decrement of the similar SPE in the following order: *σ*_(route iii)_ (1.99 × 10^−6^ S cm^−1^) > *σ*_(route iv)_ (1.56 × 10^−6^ S cm^−1^) > *σ*_(route i)_ (1.36 × 10^−6^ S cm^−1^) > *σ*_(route ii)_ (0.11 × 10^−6^ S cm^−1^) [100]. They have speculated that the electrolyte made from microwave-assisted solution-cast/hot-melt-press procedure has produced more of a pathway for ionic conduction as compared to other methods [100]. Another unrelated work has compared the usefulness of the blending approach with grafting technique on LiClO_4_-containing SPE system with sulfonated polyether ether ketone (SPEEK) and polyethylene glycol (PEG) as polymer hosts [101]. The SPEEK grafted with PEG electrolyte gave higher conductivity (around 10^−5^ S cm^−1^) when compared to SPEEK–PEG blended electrolyte (10^−6^ S cm^−1^) at 30 °C, thus implying the grafting method can suppress PEG crystallinity more than the blending technique [101]. 

Liu and co-workers [102] have combined both chemical (crosslinking) and physical (blending) routes together, i.e., crosslinked (3-glycidyloxypropyl)trimethoxysilane (GLYMO) with polyetheramine and also crosslinked poly(ethylene glycol) diglycidyl ether (PEGDGE) with polyetherdiamine before blending their products at 70:30 ratio in THF solvent and added with LiClO_4_ salt. The [EO]/[Li] ratio was optimized at 16:1. Such a unique SPE exhibited the conductivities of 1.20 × 10^−4^ S cm^−1^ and 8.30 × 10^−4^ S cm^−1^ at 30 and 80 °C, respectively [102]. The *T_g_* value was −43.6 °C, and the transference number of Li^+^ ions was 0.28 at 70 °C. This electrolyte was thermally stable up to 260 °C and gave the capacities of 110 mAh g^−1^ (The first cycle) and 61 mAh g^−1^ (100th cycle) when applied in LiFePO_4_/Li battery at 0.2 C rate [102]. As a summary, blended SPEs show superior properties than SPEs containing single polymer matrix.

#### 3.2.5. Plasticized Solid Polymer Electrolytes 

The main idea of plasticized polymer electrolyte is to improve the elasticity by lowering the *T_g_*. As stated in the previous section and Equation (3), the segmental movement of the polymer backbone contributes to the conductivity of SPEs. Increasing the segmental motion will improve the ionic conductivity and can be easily achieved by adding plasticizers into the polymer matrix. There are many types of plasticizers such as EC, PC, PEG, DMSO, diethyl carbonate (DEC), dimethyl carbonate (DMC), glycerol, gBL and DMF to name a few that affect the ionic conductivity of SPEs in different ways. Note that EC, PC, DMSO, gBL and DMF can also serve as a solvent, as listed in Table 3, while PEG of low molecular weight (M_w_) is usually used as a plasticizing agent. Plasticizers with high a dielectric constant, e.g., EC, as mentioned in the previous section, helps in salt dissociation, lowering the formation of ion pairs and ion aggregates leading to conductivity enhancement. Besides high dielectric constant, a good plasticizer should possess low viscosity to increase the mobility of ions. Furthermore, the incorporation of plasticizer in an electrolyte offers additional passage for ion transport. Some plasticized polymer electrolytes in the solid form that have been studied are tabulated in Table 6.

It is interesting to highlight that ionic liquids can play three roles, viz as plasticizer (to enhance the flexibility of polymer backbone), conducting salt (provides cation and anion) and solvent. They are non-volatile, safe (due to non-flammability), chemically and thermally stable, and are not prone to evaporation and crystallization [130]. In addition, they exhibit low melting temperature and outstanding ionic conductivity at room temperature condition, and they also have superior solvating ability. In the work of Widstrom et al. [114], increment in conductivity has been observed after incorporating ionic liquid, S_2_TFSI into PEO-LiTFSI SPE without affecting Li^+^ transference number. From Table 6, it is to be noted that N_1222_FSI is not an ionic liquid but an ionic plastic crystal that shows similar features to that of ionic liquids [115]. In contrast to conventional plasticizers, the mechanical integrity can still be retained upon addition of N_1222_FSI into the electrolyte besides reducing the polymer crystallinity and dissociating the salt. Moreover, the interfacial contact between electrolyte and electrode can be improved [115]. 

Another organic plastic crystal, i.e., succinonitrile (SN) is favored for its outstanding solvating capability in dissociating many salts including Li-based salts in battery application. As a result, conductivity is enhanced as can be observed in refs [105,112] from Table 6. Unfortunately, the plastic nature of SN deteriorates the mechanical strength of the electrolyte. Nonetheless, this can be easily avoided by preparing the electrolyte membrane in a different way. He et al. [131] prepared free-standing film containing PEGDA (M_w_ 6000 g mol^−1^), LiTFSI and SN via photopolymerization procedure using bis(2,4,6-trimethylbenzoyl)-phenylphosphine oxide (also known as Irgacure 819) as photo-initiator but without solvent. Such electrolyte which was highly amorphous with crosslinking network exhibited good ambient conductivity of 1.10 × 10^−3^ S cm^−1^, electrochemical stability window of 4.80 V with improved mechanical strength (0.24 MPa tensile strength, 84% elongation at break). Li_4_Ti_5_O_12_/Li cell employing this electrolyte gave promising performance (The first discharge capacity of 140 mAh g^−1^ at 0.2 C) with 93% capacity retention for 25 cycles [131]. On the other hand, it is evident from Table 6 that PEG of low molecular weight acted as plasticizer rather than secondary polymer in PEO-KI electrolyte which showed better conductivity [113] than PEG-free SPE even though PEG might have crosslinked with PEO. Instead, conductivity improvement is attributed to extra passageways for ion migration through PEG segmental movement. 

#### 3.2.6. Composite Solid Polymer Electrolytes 

Although addition of plasticizers in SPEs can easily improve the ionic conductivity, the mechanical strength of the SPEs is compromised. On the contrary, incorporating fillers in SPEs is known to improve the performance of SPEs not merely the ionic conductivity but also its mechanical strength since fillers can reduce the crystallinity of SPE films. SPE incorporated with an inorganic filler are termed as composite solid polymer electrolytes (CSPEs). In most CSPEs systems, the polymer is usually regarded as Lewis base if it donates its lone pair of electrons, while the filler is a Lewis acid in accepting the electrons. Fillers can be categorized into two types, i.e., active and inactive. Some examples of inactive fillers are titanium dioxide (TiO_2_), silicon dioxide (SiO_2_), alumina (Al_2_O_3_) and zirconia (ZrO_2_). The effects of fillers are varied depending on their particles size, surface nature and amount or concentration in the CSPEs. 

Tan et al. [132] investigated the effect of SiO_2_ and Al_2_O_3_ on the PMMA-based SPEs containing LiCF_3_SO_3_ salt. Addition of Al_2_O_3_ has improved the ionic conductivity by one order of magnitude from 1.36 × 10^−5^ S cm^−1^ to 2.05 × 10^−4^ S cm^−1^. However, this effect cannot be seen for CSPE containing SiO_2_ [132]. The reason behind Al_2_O_3_ showed significant improvement in conductivity may be because of the OH surface nature in alumina that provided additional coordination sites to oxygen atom in PMMA for ionic conduction. Moreover, the presence of H^+^ on the alumina surface also help in salt dissociation thus increasing the number density of ions leading to ionic conductivity improvement of the CSPE [132]. In the same work, they also reported that the amount of Al_2_O_3_ influenced the conductivity of CSPE [132]. The presence of high amount of alumina grains in CSPE may cause the polymer chain to be less mobile thus affecting the transport of ions that resulted in conductivity decrement. Nonetheless, it has been reported that the mechanical strength of CSPE was enhanced at high concentrations of filler [133]. Too much or too low content of fillers would not bring positive effect on the ionic conductivity [134]. Only at optimized concentration can lead to its improvement in the CSPE [134]. The findings on acidic surface nature of alumina that enhanced the conductivity of SPE [132] are also supported by Croce et al. [135]. Following Reference [135], it can be understood that extra pathway for ion transport was formed via hydrogen bonding when the surface of Al_2_O_3_ nanofiller (particle size 5.8 nm) was acidic. The SPE system that comprised PEO, LiCF_3_SO_3_ and acidic Al_2_O_3_ exhibited ionic conductivity of 2.10 × 10^−5^ S cm^−1^ at 30 °C. Slight reduction in conductivity (1.20 × 10^−5^ S cm^−1^ at 30 °C) was observed for Al_2_O_3_ neutral surface which may be due to weaker interactions as compared to that in acidic form [135]. On the contrary, there was no interaction between filler, anion of salt and polymer in the case of basic Al_2_O_3_ surface thus subsequently causing detrimental outcome to conductivity (7.30 × 10^−6^ S cm^−1^ at 30 °C) [135]. 

In another work, it was also observed that the addition of SiO_2_ successfully boosted the ionic conductivity for PVA-PVP-PEG-NaI electrolyte [136]. An improvement in amorphous region of the electrolyte film via the interaction of functional groups of the polymer with the Si-O-Si of the filler may be the reason for the conductivity increase. Li and Lian [137] studied the effect of SiO_2_ particle sizes (4 μm and 7 nm) in hydroxide conducting poly(acrylamide) (PAM) based SPE for alkaline batteries. The hydroxide conductivity of the SPE was amplified with the incorporation of 7 nm SiO_2_ but not for SPE containing 4 μm SiO_2_. Based on the micrographs and Raman studies, smaller size of SiO_2_ significantly helped to delay polymer crystallization [137]. Similar observation has been reported for Al_2_O_3_ where the smaller particle size prevented the formation of dendrites [138]. Other than size, the morphology of filler will also give different outcome to the CSPE. Lithium dendrites formation was minimized in the case of electrolyte containing PEO, lithium (bis trifluoromethyl)sulfate, SCN and SiO_2_ nanofibers as compared to similar electrolyte but with SiO_2_ nanoparticles [139]. It was observed that SiO_2_ aggregates was found in the latter, while the SiO_2_ nanofibers were homogeneously distributed in the electrospun CSPE as evidenced from SEM images. The CSPE of SiO_2_ nanofibers that had a 3D structure demonstrated better mechanical strength and flexibility with improved performance in lithium battery [139]. Incorporation of filler in electrolyte also yielded better interfacial contact with lithium metal electrode [140]. At 30 °C, SiO_2_ nanofibers CSPE exhibited the conductivity of 9.32 × 10^−5^ S cm^−1^ which was highest when comparing to CSPE with SiO_2_ nanoparticles (5.28 × 10^−5^ S cm^−1^) and SPE without SiO_2_ (4.72 × 10^−5^ S cm^−1^) [139]. Occasionally, an increment in cationic transference number was observed upon the addition of fillers in SPEs [3]. 

As discussed earlier, the contribution of inactive or passive fillers in ionic conductivity of SPEs is clear, i.e., improving the mobility of charge carriers either through the mobility of polymer chain (amorphousness) or by providing the additional transportation sites. Active fillers, such as Li_1+x_Al_x_Ge_2−x_(PO_4_)_3_ [141], Li_1+x_Al_x_Ti_2−x_(PO_4_)_3_ [142], Li_7_La_3_Zr_2_O_12_ [143], Li_0.33_La_0.557_TiO_3_ [144] and Li_6.4_La_3_Zr_1.4_Ta_0.6_O_12_ [145] on the other hand, contribute directly to the lithium-ion conductivity apart from impeding crystallization and improving the mechanical integrity of the SPEs. These materials by their own serve as solid electrolytes [146,147,148,149,150]. Nonetheless, the fabrication processes of these ceramics are usually complicated and costly [139]. In addition, the thickness of these solid electrolytes have to be large (around 100 μm in pellet form), since they are fragile and brittle [139] thus resulting in high interfacial resistance. These drawbacks lead to introducing them in SPEs as fillers instead. Filler-added SPEs are flexible, more stable and have better interfacial compatibility with Li metal electrodes [141,151]. Furthermore, the SPEs possess better processability and higher ionic conductivity than the solid electrolytes [151,152]. Similar to inactive fillers, the size, concentration and morphology of active fillers impose different impact on the CSPEs.

Among the inorganic active fillers, Li_7_La_3_Zr_2_O_12_ (LLZO) having garnet structure is favored due to its chemical stability against lithium metal and large electrochemical window (>5 V) [143]. LLZO nanofibers prepared via the electrospinning method have been introduced to an electrolyte comprising PVDF–HFP as a polymer host, LiTFSI salt and ionic liquid plasticizer [153]. The CSPE delivered high ambient conductivity (6.50 × 10^−3^ S cm^−1^), reduced crystallinity and wide electrochemical window (5.3 V) than plasticized SPE without LLZO as filler [153]. Comparison was made with basic SPE configuration of PVDF-HFP-LiTFSI in electrochemical performance of Li-ion battery. The LLZO CSPE cell displayed good cyclability over 1000 cycles with specific capacity of 149 mAh g^−1^, whereas the capacity decreased sharply after 50th cycle for the Li-ion cell with basic SPE [153]. Synergistic effect of LLZO filler and poly(ethylene glycol) dimethyl ether (PEGDME) plasticizer was also studied in PEO–LiTFSI SPEs system [154]. Again, improvements in conductivity, Li-ion transference number and electrochemical performance were reported [154]. In the work of Keller et al. [155], LLZO-added CSPE containing PEO and LiTFSI did not contribute to ionic conductivity but exhibited improved electrolyte-electrode interfacial contact and higher electrochemical performance in lithium battery. It was found that the addition of LLZO in SPEs suppressed lithium dendrites growth [152,153,154,155,156]. The lithium and zirconium sites of LLZO can be doped with other elements to boost its properties. Zhang and co-authors [157] employed tantalum (Ta) as dopant in LLZO to form Li_6.75_La_3_Zr_1.75_Ta_0.25_O_12_ (LLZTO). The PAN-LiClO_4_-LLZTO CSPE showed conductivity increment by 12 times (2.20 × 10^−4^ S cm^−1^ at 40 °C) and higher Li^+^ transference number of 0.30 than filler-free SPE [157]. In addition, the CSPE was thermally and electrochemically stable with enhanced mechanical strength [157]. It is worthy to mention that Li^+^ transference number as high as 0.75 was obtained in CSPE containing LLZTO, poly(propylene carbonate) and LiTFSI with conductivity of 5.20 × 10^−4^ S cm^−1^ at 20 °C [158]. It is understood that the addition of nano-sized LLZTO promoted salt dissociation due to the interactions between polymer, cation and filler and subsequently impeded the diffusion of anions [158]. 

LLZO is preferred over NASICON-type Li_1.5_Al_0.5_Ge_1.5_(PO_4_)_3_ (LAGP) due to their inexpensive elements [155]. In contrast, germanium in the latter is costly [155]. Nevertheless, LLZO has shortcomings; for example, it has an unstable at room-temperature condition, and thus, it is reactive towards moisture and humidity [155]. Similar to LLZO, LAGP is also unreactive towards lithium metal electrodes and has been used as a filler in polymer electrolytes. PEO, LiTFSI and LAGP were mixed and ground without solvent before heating and hot-pressing to form CSPE as reported by Piana et al. [159]. The CSPE showed improvements in term of conductivity, mechanical integrity and specific capacity as compared to electrolyte without filler [159]. On the other hand, the prepared CSPE in Reference [159] showed superior interfacial contact than pelletized LAGP solid electrolyte in lithium-ion cells. However, no significant change in lithium-ion transference number was observed with the introduction of LAGP in PEO-based electrolyte. On the contrary, LAGP added in poly(propylene carbonate)-based electrolyte increased lithium-ion transference number to 0.77 with conductivity of 0.56 × 10^−3^ S cm^−1^ and improved cyclability and stability in Li-ion battery [160]. 

Li et al. [13] employed electrospinning technique to synthesize Li_0.33_La_0.557_TiO_3_ nanorodsbefore adding in PVDF–HFP electrolyte containing LiTFSI salt. The CSPE was more thermally stable and showed decreased crystallinity with higher ambient ionic conductivity (1.21 × 10^−4^ S cm^−1^) and tensile strength (39.77 MPa) than filler-free electrolyte (*σ* = 4.72 × 10^−5^ S cm^−1^; tensile strength = 38.41 MPa) [13]. Ionic conductivity of PAN-based SPE has been shown to increase with the help of an active filler namely lithium titanate nanotubes (LiTNT) [161]. From the vibrational studies, Pignanelli and co-workers observed that the presence of LiTNT in SPE helped in the increment of lithium perchlorate dissociation, whereas the impedance spectroscopy showed two semicircles that are related to lithium-ion conductivity [161]. The semicircle in the higher frequency region is accredited to the bulk resistance of lithium transport across the polymer matrix, whereas the second is ascribed to lithium transport through the interaction with the LiTNT [161]. Some authors have proposed that lithium transport can take place via three possible routes, i.e., polymer—salt matrix, polymer—filler interface and inside filler grain [162,163]. Zheng et al. [163] showed that Li-ion movement took place mainly via ceramic filler LLZO grain for PEO-LiClO_4_-LLZO system, using NMR results. On the other hand, for PEO-LiClO_4_-TEGDME-LLZO electrolyte, it was found that Li-ion motion via TEGDME-related phase was preferred over filler or polymer matrix or polymer—filler interface [164]. In our opinion, a complete understanding on the conduction mechanism of such SPEs remain challenging as there are many influential factors behind it. Other than the abovementioned ceramic fillers, Li_6.4_La_3_Zr_2_Al_0.2_O_12_ and LiSnZr(PO_4_)_3_ have also been incorporated into PEO-LiTFSI [165] and PVDF–LiTFSI [166] electrolytes, respectively. 

Another category of active fillers in CSPEs are the metal organic frameworks (MOFs) which display excellent properties such as microporous structures with high surface area, good mechanical and thermal properties, and controllable pore structure. MOFs have been credited to an increased lithium-ion transference number, ionic conductivity and improve the cycling stability of batteries [167]. Several examples of MOFs as active fillers in CSPEs for battery application include aluminum(III)-1,3,5-benzenetricarboxylate (Al-BTC) [168], D-UiO-66-NH_2_ [169], copper-1,4-benzenedicarboxylate (Cu-BDC) [170], HKUST-1(Cu) [171] and magnesium-benzene tricarboxylate (Mg-BTC) [172] to name a few. Overall, MOFs having 3D framework and controllable pore structures improve the interfacial contact between electrode and electrolyte. This is reflected with the addition of Zn_4_O(1,4-benzenedicarboxylate) in PEO-LiTFSI electrolyte which stabilized the interfacial resistance and improved the cyclability up to 100 cycles [173]. As usual, its ionic conductivity was enhanced too [173]. CSPE having Cu-BDC showed enhanced thermal stability which led to safer battery at high temperatures as compared to filler-free SPE [170]. More detailed reviews on CSPEs are available elsewhere [151,174,175,176]. Some other CSPEs systems that have been reported by many researchers are tabulated in Table 7.

#### 3.2.7. SPEs Containing Other Kind of Additives 

It is an undeniable fact that ionic conductivity plays a crucial role in ensuring the performance of electrochemical cells, but the cationic transference number also influences the efficacy of devices in particular batteries. Enhancement in lithium-ion transference number has been observed in PEO-LiI SPEs upon the addition of calix [4] arene with values around 0.8 to 1 at elevated temperatures between 55 and 90 °C [189]. In most cases, the cationic transference number values are found to be below 0.4 without supramolecular additives in PEO-salt SPE systems as mentioned earlier. Based on ab initio computational work, Johansson [190] predicted that the cryptands, i.e., organic compounds having large 3D molecular structure, can trap anions, such as Cl^−^, ClO_4_^−^, BF_4_^−^ and F^−^, and form coordination complexes, thereby reducing their tendency to re-associate with Li^+^ cation and consequently increasing the cationic transference number. However, the supramolecular compounds are unable to confine large PF_6_^−^, TFSI^−^ or [(CF_3_SO_2_)_2_N]^−^ anions within a 3D network and cannot form complexes [190].

Mazor et al. [191] investigated the influence of calix[6]pyrrole on PEO-LiCF_3_SO_3_ SPEs. Although the ambient conductivity was found to decrease with the addition of calix, the conductivity at 60 °C was higher than that without calix. Nevertheless, the *t_Li_*_+_ of the calix-added SPE enhanced by 185% to 0.74 from 0.26 of the calix-free SPE at 60 °C [191]. Ambient conductivity decrement is said to be attributed to the increased rigidity of polymer network, whereas an improvement in lithium-ion transference number is due to increased amount of free ions from dissociated ion aggregates. The SPE has been employed in Li/MoO_x_S_y_ cells that exhibited a power level of 2.1 mW cm^−2^ at 90 °C and a discharge capacity of ~24 μAh on the 60th cycle [191]. It is said that calix[6]arene can minimize the growth of lithium dendrites and subsequently improves cell performance [192]. A similar observation wherein an increased lithium-ion transference number (*t_Li_*_+_ = 0.95 at 70 °C) was accompanied by conductivity decrement has been reported in PEO-LiBF_4_-calix[6]pyrrole system [193]. In addition, the lithium diffusion-coefficient values were 2.0 × 10^−7^ cm^2^ s^−1^ and 2.5 × 10^−7^ cm^2^ s^−1^ for calix-free and calix-incorporated PEO-based electrolytes, respectively [193].

As aforementioned, the incorporation of supramolecular additives, such as calix-based compounds, is used to increase cationic transference number by trapping/immobilizing anion of the salt [194]. Usually, this will lead to reduce the anionic mobility and unwittingly decrease the ionic conductivity, as stated in preceding paragraph. Nevertheless, there are some controversial reports that showed otherwise. Won and co-workers [195] observed that the SPE containing PVC doped with lithium chloride (LiCl) salt and calix[4]pyrrole exhibited higher ionic conductivity, at around 10^−3^ S cm^−1^ at 25 °C, compared to that calix-free SPE (10^−5^ S cm^−1^). Using density functional theory, they obtained that the dissociation energies for LiCl-calix and PVC-LiCl-calix complexes were 110.8 and −18.1 kcal mol^−1^, whereas, for LiCl salt, the dissociation energy value was 145.6 kcal mol^−1^ [195]. A lower dissociation energy value implies that the cation of salt is more free to move, while its anion has complexed with calix [195].

Plastic crystal SN has also been employed as a polymer matrix in electrolyte, besides serving as a plasticizer and ionizer for both Li- and Na-based salts [131,196]. In order to improve the transference number of Na^+^, Chen et al. [196] synthesized boron-containing 2-hydroxyethyl methacrylate (B-HEMA) before incorporating it into SN-based polymer electrolyte that had non-woven polypropylene-cellulose composite framework and NaClO_4_ salt. This electrolyte having B^-^ (boron anion) acceptor was prepared via a UV-assisted curing method, using 2-hydroxy-2-methyl-1-phenyl-1-propanone as photo-initiator. This electrolyte demonstrated superior properties in terms of ambient conductivity (3.60 × 10^−4^ S cm^−1^), tensile strength (28.2 MPa) and Na^+^ transference number of (*t_Na+_* = 0.62) [196]. Promising results were also obtained after applying the electrolyte in Na-ion batteries (initial capacity of ~105 mAh g^−1^ at 0.1 C and retaining 80% capacity after 120 cycles) [196]. The boron moiety is said to complex with ClO_4_^-^ ion of the salt, thus lowering the anionic mobility and subsequently enhancing the Na^+^ ion transference number. Meanwhile, the authors have stated that the polypropylene–cellulose structure did not contribute in ionic conduction, but instead improved the mechanical property of the plastic crystal polymer electrolyte [196].

Polyaniline (PANI), which is a conducting polymer that is usually used as counter electrode in DSSCs [197,198], has been incorporated in SPE containing PEO, tetrapropylammonium iodide (TPAI) and I_2_ to trap the large cations ((C_3_H_7_)_4_N^+^ or simply TPA^+^) so that the anions can contribute more to conduction, which is beneficial in DSSC [199]. The authors also believed that PANI has performed the role of plasticizing agent in decreasing crystallinity of PEO and its melting point based on DSC analysis. Upon PANI addition, the degrees of crystallinity and *T_m_* were reduced to 3.37% and 66.2 °C from 8.93% and 66.5 °C [199]. The PANI-added SPE attained higher values of room temperature conductivity (8.61 × 10^−5^ S cm^−1^) and ionic transference number (0.99) than PANI-free SPE (*σ* = 6.81 × 10^−5^ S cm^−1^; *t_ion_*= 0.96). The former also gave better DSSC performance (5.01% efficiency) than the latter (efficiency 3.5%) [199].

A special additive, i.e., hydroxypropyl trimethylammonium bis(trifluoromethane) sulfonamide chitosan salt (HACC-TFSI), was synthesized via ion-exchange route, using hydroxypropyl trimethylammonium chloride chitosan salt and LiTFSI before HACC-TFSI, PEO and LiTFSI were mixed in DMF solvent to form SPE via the solution-casting technique [200]. Ionic conductivity values for HACC-TFSI-added PEO electrolyte at 30 and 60 °C were 1.77 × 10^−5^ S cm^−1^ and 5.01 × 10^−4^ S cm^−1^, respectively [200]. In contrast, conductivities of 3.80 × 10^−6^ S cm^−1^ and 1.90 × 10^−4^ S cm^−1^ were recorded at the same temperature by PEO SPE without HACC-TFSI [200]. Besides better conductivity, Li^+^ ion transference number and tensile strength increased by 1.7 and 3.6 times. Moreover, the SPE with HACC-TFSI was found to be resistant against elevated temperature (150 °C) by retaining its shape after exposure at high temperature for one hour [200]. On the contrary, the electrolyte without HACC-TFSI has melted under similar conditions. LiFePO_4_/Li cell with HACC-TFSI added SPE demonstrated good cyclability and stability over 100 cycles maintaining 73% capacity of its initial capacity of ~108 mAh g^−1^ [200]. It is worthy to mention that this is considered quite an achievement, since there are not many cells with SPEs that can withstand and operate under such a high temperature.

## 4. Summary and Outlook

Solid-state electrochemical devices are the focus for the next-generation energy sources, especially for large power-consumption machines, such as electric vehicles. SPEs in electrochemical devices offer flexibility, low cost, harmless and cell cycling improvement. Basic SPEs which contain polymer and salt typically exhibit the ambient ionic conductivity of ~10^−5^ S cm^−1^. The conductivity must be improved at least to 10^−3^ S cm^−1^ for application in devices. Additives such as plasticizers and fillers are able to improve the conductivity of the SPEs. Plasticizers are able to reduce the crystallinity of the SPEs and enhance the elasticity and the segmental motion of the polymer chain, leading to an increase the overall ionic conductivity. Fillers, on the other hand, contribute either directly (active) or indirectly (inactive) towards conductivity enhancement. In contrast to SPEs with the plasticizers, fillers are able to improve the mechanical strength of the SPEs. Blending polymers is another approach that is used specially to improve the mechanical strength, apart from conductivity enhancement. Although ionic conductivity is one significant factor determining the feasibility of SPEs in electrochemical devices, other factors, such as ionic transport and transference number of ions, also influence the performance. It is appreciable that various efforts have been undertaken in developing suitable SPEs for practical use in electrochemical devices. In our opinion, there is a promising future for SPEs to compete and substitute liquid electrolytes in electrochemical devices. Among the abovementioned approaches, we think that there is much room for developments and improvements on SPEs having active fillers, in particular, MOFs, since research on this is relatively new and less than the others. Moreover, so far, the research only focuses on MOF-containing SPEs in application for Li-ion batteries. We believe that SPEs containing MOFs as fillers have the potential to be applied in other electrochemical devices, including Li-air batteries, DSSCs, PEMFCs and supercapacitors.

## Figures and Tables

**Table 1:** Typical polymers used in polymer electrolytes, along with their characteristics.

Polymer	Functional Group(s)	Glass Transition Temperature, *T_g_* (°C)	Reference
Poly(ethylene oxide) (PEO)	Ether	−64	[19]
Polyacrylonitrile (PAN)	Nitrile	125	[19,20]
Poly(vinyl alcohol) (PVA)	Hydroxyl	82	[21]
Cellulose	Hydroxyl, Ether	220	[22]
Poly(methyl methacrylate) (PMMA)	Ester, Carbonyl	105	[19]
Polyethyl methacrylate (PEMA)	Ester, Carbonyl	63	[23]
Polyvinylidene fluoride (PVDF)	Difluoromethylene	−40	[19]
Chitosan	Hydroxyl, Amine, Ether	203	[24]
Poly(vinylidene fluoride-hexafluoropropylene) (PVDF-HFP)	Difluoromethylene	−90	[19]
Starch	Hydroxy, Ether	70	[25]

**Table 2:** Some popular salts used in polymer electrolytes, along with their properties [18,26,27].

Salt	Cationic Radii (pm)	Anionic Radii (pm)	Lattice Energy (kJ mol^−1^)
Lithium bis(trifluoromethanesulfonyl)imide ((LiN(CF_3_SO_2_)_2_) or (LiTFSI)	60	328	564
Lithium trifluoromethanesulfonate or lithium triflate (LiCF_3_SO_3_)	60	268	730
Lithium perchlorate (LiClO_4_)	60	234	718
Lithium tetrafluoroborate (LiBF_4_)	60	227	735
Lithium hexafluorophosphate (LiPF_6_)	60	254	681
Sodium iodide (NaI)	102	216	674
Sodium thiocyanate (NaSCN)	102	213	680
Ammonium iodide (NH_4_I)	143	216	605
Lithium iodide (LiI)	60	216	762

**Table 3:** Physical properties of some solvents [28,29,30].

Solvent	Donor Number, DN	Dielectric Constant, *ε* at 25 °C	Viscosity (cP) at 25 °C	Melting Point, *T_m_* (°C)	Boiling Point (°C)
Acetone	17.0	20.6	0.30	−94.7	56.1
Acetonitrile (ACN)	14.1	35.9	0.37	−43.8	81.6
N-methyl-2-pyrrolidone (NMP)	27.3	32.2	1.67	−24.4	202
Tetrahydrofuran (THF)		7.58	0.46	−108.4	60
N,N-Dimethylacetamide (DMAc)	27.8	37.8	0.93	−20	166.1
Dimethyl sulfoxide (DMSO)	29.8	46.5	1.99	18.5	189
Gamma-butyrolactone (gBL)	18.0	39.0 ^†^	1.70	−44	204
N,N-Dimethylformamide (DMF)	26.6	36.1	0.80	−61	153
Ethylene carbonate (EC)	16.4	89 *	1.90 *	36	238
Propylene carbonate (PC)	15.1	64	2.50	−49	241
Ethanol	19.2	25	1.08	−114	78
Water	18.0	78	0.89	0	100

* At 40 °C. ^†^ At 20 °C.

**Table 4:** Basic SPEs films, along with their conductivity and significant properties.

Electrolyte System	Preparation Method	Conductivity, *σ* (S cm^−1^)	Important Findings	Reference
PEO-NaSCN	Solution casting	9.86 × 10^−8^ (RT)	*t_ion_* = 0.85 *n* = 1.21 × 10^20^ m^−3^; *μ* = 5.10 × 10^−7^ m^2^ V^−1^ s^−1^	[36]
PEO-NaFNFSI [EO]/[Na]=15	Solution casting	3.16 × 10^−6^ (80 °C) 3.36 × 10^−4^ (30 °C)	Thermal stability > 300 °C; *T_g_* = −36.3 °C *t*_Na+_ = 0.24 (80 °C) Oxidation potential = 4.87 V vs Na^+^/Na NaCu_1/9_Ni_2/9_Fe_1/3_Mn_1/3_O_2_/SPE/Na cell: Initial capacity = 122.4 mAh g^−1^ (0.1 C, 80 °C)70% capacity retention at 1 C after 150 cycles (80 °C)	[37]
PEO-LiCF_3_SO_3_	Ball-milling and hot press	1.00 × 10^−6^	*T_g_* = −64.4 °C; %χ_C_ = 37.3%	[38]
PAN-LiTFSI	Solution casting	2.54 × 10^−4^ (25 °C)	%χ_C_ = 9.3% ITO-WO_3_/SPE/CeO_2_-TiO_2_-ITO ECD: Cathodic coloration at −1.25 V; Anodic bleaching at −0.40 V	[39]
CMC-(NH_4_)_2_CO_3_	Solution casting	7.71 × 10^−6^ (30 °C)	%χ_C_ = 30.9%; *t_ion_* = 0.98 CMC film without salt: *σ* = 10^−9^ S cm^−1^	[40]
NaCMC-Na_2_S-S	Solution casting	2.79 × 10^−5^ (30 °C)	*E_A_* = 0.38 eV *n* = 15.24 × 10^18^ cm^−3^; *μ* = 11.44 × 10^−6^ m^2^ V^−1^ s^−1^, *D* = 2.94 × 10^−7^ cm^2^ s^−1^ TiO_2_-CdS-ZnS/SPE/Pt QDSSC (light intensity of 100 mW cm^−2^): OCV = 0.41 V; *J_sc_* = 4.92 mA cm^−2^; *FF* = 0.45; *η* = 0.90%	[41]
Pectin-LiCl	Solution casting	1.96 × 10^−3^ (30 °C)	*E_A_* = 0.23 eV Tensile strength = 4.61 MPa Electrochemical stability window 3.77 V Zn/SPE/LiFePO_4_ cell: OCV = 1.25 V	[42]
PEO-LiCF_3_SO_3_ [EO]/[Li] = 9:1	Solution casting	1.40 × 10^−6^ (30 °C)	*T_g_* = −39 °C; *T_m_* = 64 °C	[43]
Sago starch-KI	Solution casting	3.41 × 10^−4^ (RT)	Dielectric relaxation time = 2.33 × 10^−7^ s N3/TiO_2_/SPE/Pt DSSC (light intensity of 100 mW cm^−2^): OCV = 0.58 V; *J_sc_* = 0.29 mA cm^−2^; *FF* = 0.43; *η* = 0.57%	[44,45]
Corn starch-MgSO_4_	Solution casting	8.52 × 10^−5^ (30 °C)	SEM micrograph of SPE shows rough surface, while that of starch film without salt shows otherwise.	[46]
PEMA-NH_4_I	Solution casting	1.80 × 10^−5^ (30 °C)	*t_ion_* = 0.93 Ru-based dye/TiO_2_/SPE/Pt DSSC (light intensity of 100 mW cm^−2^): OCV = 0.56 V; *J_sc_* = 1.52 mA cm^−2^; *FF* = 0.51; *η* = 0.43%	[47]

NaSCN—sodium thiocyanate; RT—room temperature; NaFNFSI—sodium (fluorosulfonyl)(n-nonafluorobutanesulfonyl)imide (Na[(FSO_2_)(n-C_4_F_9_SO_2_)N]; %χ_C_—degree of crystallinity percentage; CMC—carboxymethyl cellulose; ITO—indium tin oxide; WO_3_—tungsten (VI) oxide; CeO_2_—cerium (IV) oxide; TiO_2_—titanium dioxide; (NH_4_)_2_CO_3_—ammonium carbonate; NaCMC—sodium-carboxymethylcellulose; Na_2_S—sodium sulfide; S—sulfur; OCV—open circuit voltage; *J_sc_*—short circuit current density; *FF*—fill factor; *η*—efficiency; CdS—cadmium sulfide; ZnS—zinc sulfide; Pt—platinum; LiCl—lithium chloride; LiFePO_4_—lithium iron phosphate; N3—*cis*-bis(isothiocyanato)bis(2,2′-bipyridyl-4,4′-dicarboxylato)ruthenium(II); MgSO_4_—magnesium sulfate.

**Table 5:** Various blended SPEs systems, along with their conductivity values and other important parameters.

Electrolyte System	Preparation Method	Conductivity, *σ* (S cm^−1^)	Important Findings	Reference
PVA:PVP (70:30)-25 wt.% LiNO_3_	Solution casting	6.83 × 10^−4^	*E_A_* = 0.27 eV; *T_g_* = 80 °C 70 PVA:30 PVP film: *σ* = 1.58 × 10^−6^ S cm^−1^; *T_g_* = 130 °C Degree of swelling lower than blend SPE	[84]
Chitosan:PVA (1:1)-NH_4_I	Solution casting	1.77 × 10^−6^ (30 °C)	*E_A_* = 0.38 eV Without PVA blend: *σ* = 3.73 × 10^−7^ S cm^−1^ (30 °C) *E_A_* = 0.46 eV	[85]
Chitosan:PEO (1:1)-NH_4_I	Solution casting	3.66 × 10^−6^ (30 °C)	*E_A_* = 0.31 eV; *t_ion_* = 0.85 N3/Ag-TiO_2_/SPE/Pt DSSC (light intensity of 100 mW cm^−2^): OCV = 0.58 V; *J_sc_* = 2.84 mA cm^−2^; *FF* = 0.69; *η* = 1.13% Without PEO blend: σ = 3.73 × 10^−7^ S cm^−1^ (30 °C); *E_A_* = 0.46 eV	[86]
PEMA:PVDF-HFP (70:30)-LiCF_3_SO_3_	Reflux + Solution casting	2.87 × 10^−7^ (RT)	PEMA-LiCF_3_SO_3_: σ = 9.18 × 10^−8^ S cm^−1^ (RT)	[87]
50% (PEO+LiTFSI):50% PPC [EO]/[Li] = 18:1	Solution casting	2.04 × 10^−5^ (25 °C) 2.82 × 10^−4^ (60 °C)	*E_A_* = 0.78 eV; *T_g_* = −41.3 °C; *T_m_* = 49.7 °C; %χ_C_ = 22.10%; *t_Li_*_+_ = 0.184 (60 °C); Electrochemical stability window 4.90 V LiFePO_4_/SPE/Li cell (0.5C, 60 °C): Discharge specific capacity of 112 mAh g^−1^ after 100th cycle Without PPC blend: σ = 1.02 × 10^−5^ S cm^−1^ (25 °C); σ = 1.77 × 10^−4^ S cm^−1^ (60 °C); *E_A_* = 0.80 eV; *T_g_* = −35.2 °C; *T_m_* = 58.1 °C; %χ_C_ = 48.07%; *t_Li_*_+_ = 0.156 (60 °C); Electrochemical stability window 4.25 V Discharge specific capacity = 93 mAh g^−1^ after 100th cycle (0.5 C, 60 °C) Decreased crystallinity with PPC addition Improved interface stability	[88]
PEO:PVP (9:1)-8 wt.% LiClO_4_	Solution casting	0.23 × 10^−5^ (30 °C)	Bandgap energy = 4.07 eV (indirect) and 4.35 eV (direct) Thermally stable till 324 °C Lowest intensity in photoluminescence results compared to that at other PEO:PVP ratios	[89,90]
CMC:PVA:NH_4_NO_3_ (56:14:30 wt.%)	Solution casting	1.70 × 10^−3^	*T_g_* = 88.7 °C; *t*_H+_ = 0.42 Decomposition temperature = 340 °C	[91]
MG49:PMMA (70:30)-25 wt.% LiBF_4_ [O]/[Li] = 5:1	Solution casting	8.60 × 10^−6^ (30 °C)	MG49-PMMA film: σ = 1.10 × 10^−12^ S cm^−1^ (30 °C)	[73]
MG49:PMMA (70:30)-25 wt.% LiClO_4_ [O]/[Li] = 6:1	Solution casting	1.50 × 10^−8^ (30 °C)	MG49-PMMA film: σ = 1.10 × 10^−12^ S cm^−1^ (30 °C)	[73]
CMKC:CMC (60:40)-LiI	Solution casting	3.89 × 10^−3^ (27 °C)	*T_g_* = −43.0 °C N719/TiO_2_/SPE/Pt DSSC (light intensity of 100 mW cm^−2^): OCV = 0.492 V; *J_sc_* = 0.40 mA cm^−2^; *FF* = 0.57; *η* = 0.11%	[92]
HCh:PVC (90:10)-NaI	Solution casting	1.50 × 10^−5^ (RT)	%χ_C_ = 24%; *D* = 1.10 × 10^−8^ cm^2^ s^−1^; *μ* = 4.10 × 10^−7^ cm^2^ V^−1^ s^−1^; *n* = 2.30 × 10^20^ cm^−3^ N3/TiO_2_/SPE/Pt DSSC (light intensity of 100 mW cm^−2^): OCV = 0.58 V; *J_sc_* = 8.62 mA cm^−2^; *FF* = 0.59; *η* = 2.93%	[93]
PEO:WPU (3:1)-LiTFSI [EO]/[Li] = 16:1	Solution casting	3.10 × 10^−3^ (80 °C)	*T_g_* = −45.4 °C; thermally stable till 300 °C LiFePO_4_/SPE/Li battery: Discharge capacity = 122 mAh g^−1^ after 100th cycle (1C, 80 °C), 96% capacity retention	[94]
PEO:TPU (3:1)-LiTFSI [EO]/[Li] = 16:1	Solution casting	5.30 × 10^−4^ (60 °C)	*t_Li_*_+_ = 0.31; Tensile stress 1.38 MPa LiFePO_4_/SPE/Li battery: Discharge capacity = 112 mAh g^−1^ after 100th cycle (1 C, 60 °C), 96% capacity retention	[95]
PEO-PVP-NaIO_4_ (65:25:10 wt.%)	Solution casting	1.56 × 10^−7^ (30 °C)	*E_A_* = 0.22 eV; *T_g_* = 50.2 °C; %χ_C_ = 55.0% D = 2.72 × 10^−8^ cm^2^ s^−1^; *μ* = 1.0 × 10^−4^ cm^2^ V^−1^ s^−1^; *n* = 9.35 × 10^15^ cm^−3^ Direct optical bandgap = 3.60 eV Refractive index = 2.28	[96]
PEO:PVP (4:1)-Mg(NO_3_)_2_	Solution casting	5.80 × 10^−4^ (25 °C)	*E_A_* = 0.31 eV; *t*_+_ = 0.33 Thermally stable till 220 °C MgMn_2_O_4_/SPE/Mg battery: OCV = 1.46 V	[97]
PVDF-HFP:PEO (4:1)-KI-TBAI-I_2_	Solution casting	4.53 × 10^−5^ (25 °C)	*T_m_* = 144.9 °C; %χ_C_ = 18.1% N3/TiO_2_/SPE/Pt DSSC (light intensity of 60 mW cm^−2^): OCV = 0.674 V; *J_sc_* = 4.39 mA cm^−2^; *FF* = 0.50; *η* = 2.46% Without PEO blend: *σ* = 9.99 × 10^−6^ S cm^−1^ (25 °C) *T_m_* = 146.5 °C; %χ_C_ = 23.5% OCV = 0.648 V; *J_sc_* = 3.78 mA cm^−2^; *FF* = 0.46; *η* = 1.88%	[98]
PVAc:PMMA (70:30)-LiCl	Solution casting	1.03 × 10^−5^ (30 °C)	*E_A_* = 0.25 eV; *T_g_* = 42.6 °C; *t_+_* = 0.97 *D*_+_ = 6.93 × 10^−11^ cm^2^ s^−1^; *μ* = 2.65 × 10^−9^ cm^2^ V^−1^ s^−1^; *n* = 2.35 × 10^22^ cm^−3^	[99]

PVP—poly(vinyl pyrrolidone); LiNO_3_—lithium nitrate; PPC—propylene carbonate; CMKC—carboxymethyl κ-carrageenan; N719—di-tetrabutylammonium cis-bis(isothiocyanato)bis(2,2′-bipyridyl-4,4′-dicarboxylato) ruthenium(II); PVC—poly(vinyl chloride); WPU—waterborne polyurethane; TPU—thermoplastic polyurethane; NaIO_4_—sodium periodate; Mg(NO_3_)_2_—magnesium nitrate; TBAI—tetrabutylammonium iodide; PVAc—poly(vinyl acetate); LiCl—lithium chloride.

**Table 6:** Examples of plasticized SPEs systems found in the literature.

Electrolyte System	Preparation Method	Conductivity, *σ* (S cm^−1^)	Important Findings	Reference
PEO-LiPF_6_-EC	Solution casting	2.06 × 10^−4^ (25 °C)	Bandgap energy = 5.63 eV (indirect) and 5.80 eV (direct) Unplasticized SPE: *σ* = 4.10 × 10^−5^ S cm^−1^ (25 °C) Bandgap energy = 5.74 eV (indirect) and 5.90 eV (direct)	[103]
PGMA-LiClO_4_-EC	Solution casting	2.00 × 10^−4^ (RT)	PGMA was synthesized via photo-polymerization route. *E_A_* = 0.21 eV Electrochemical stability window 3.00 V Unplasticized SPE: *σ* = 4.20 × 10^−5^ S cm^−1 ^(RT); *E_A_* = 0.26 eV Electrochemical stability window 2.50 V	[104]
PEO-LiClO_4_-SN	Solution casting	~9.12 × 10^−5^ (20 °C)	*E_A_* = 0.45 eV Unplasticized SPE: *σ* = 3.79 × 10^−7^ S cm^−1^ (20 °C) *E_A_* = 0.89 eV	[105]
HEC-LiCF_3_SO_3_-glycerol [EO]/[Li] = 6:1	Solution casting	1.06 × 10^−5^ (30 °C)	*E_A_* = 0.18 eV; *T_g_* = −83 °C	[106]
Chitosan-LiClO_4_-glycerol	Solution casting	~3.00 × 10^−5^ (25 °C)	Too much glycerol, SPE easily torn off. Without glycerol: *σ* = 1.40 × 10^−5^ S cm^−1^ (25 °C)	[107]
Chitosan-LiClO_4_-glycerol	Solution casting	1.20 × 10^−3^ (25 °C)	*t_io_*_n_ = 0.955 *D* = 4.19 × 10^−9^ cm^2^ s^−1^; *μ* = 1.63 × 10^−7^ cm^2^ V^−1^ s^−1^; *n* = 4.57 × 10^22^ cm^−3^ Specific capacitance = 98.99 F g^−1^ at scan rate 10 mV s^−1^	[108]
PVB-LiClO_4_-PEG_400_	Solution casting	2.15 × 10^−6^ (RT)	Unplasticized SPE: *σ* = 5.80 × 10^−10^ S cm^−1^	[109]
PEMA-NH_4_I-EC	Solution casting	1.16 × 10^−5^ (RT)	*t_ion_* = 0.85 *n* = 1.75 × 10^21^ m^−3^	[110]
PEMA-NH_4_CF_3_SO_3_-BMATSFI	Solution casting	8.35 × 10^−4^ (30 °C)	*T_g_* = 2 °C; *t_ion_* = 0.82 Unplasticized SPE: *σ* = 1.02 × 10^−5^ S cm^−1^; *T_g_* = 68 °C	[111]
PVDF-HFP-LiClO_4_-SN	Solution casting	~1.62 × 10^−3^ (20 °C)	*E_A_* = 0.40 eV Unplasticized SPE: *σ* = ~6.38 × 10^−9^ S cm^−1^ (20 °C) *E_A_* = 0.69 eV	[105]
PVDF-HFP-LiBETI-SN	Solution casting	~2.58 × 10^−3^ (20 °C)	LiCoO_2_/SPE/Li_4_Ti_5_O_12_ cell (C/10, 25 °C): Initial discharge capacity 116 mAh g^−1^, 78% capacity retention after 120th cycle	[105]
PVDF-PEO-LiClO_4_-SN	Solution infiltration in PVDF film + drying	2.80 × 10^−5^(25 °C)	*T_g_* = −75.6 °C; *t_Li_*_+_ = 0.37 (25 °C) LiFePO_4_/SPE/Li: OCV = 3.1 V, discharge capacity 109 mAh g^−1^ (100th cycle), 90% capacity retention	[112]
PEO-LiCF_3_SO_3_-TiO_2_-EC [EO]/[Li] = 9:1	Solution casting	1.60 × 10^−4^ (30 °C)	*E_A_* = 0.60 eV; *T_g_* = −50 °C; *T_m_* = 50 °C	[43]
PEO-KI-PEG	Solution casting	5.27 × 10^−5^ (25 °C)	Unblended PEO-KI SPE: *σ* = 1.96 × 10^−5^ S cm^−1^ (25 °C) Low M_w_ of PEG (~4000) might be trapped or crosslink in PEO-KI network	[113]
PEO-LiTFSI-S_2_TFSI	Mixing, vacuum-sealed in pouch and hot press	0.96 × 10^−3^ (22 °C) 4.00 × 10^−3^ (60 °C)	*t_Li_*_+_ = 0.31 (60 °C) LiFePO_4_/SPE/Li: Discharge capacity (1 C, 60 °C) = 160.1 mAh g^−1^ (500th cycle), 89.9% capacity retention	[114]
PEO-LiFSI-N_1222_FSI [EO]/[Li] = 20:1	Solution casting	1.78 × 10^−5^ (25 °C) 2.14 × 10^−4^ (50 °C)	*T_g_* = −58.1 °C; %χ_C_ = 24.7% High thermal stability with decomposition temperature above 220 °C LiFePO_4_/SPE/Li: Discharge capacity (0.2 C, 50°C) = 151.5 mAh g^−1^ (120th cycle), 95.4% capacity retention Unplasticized SPE: *σ* = 7.24 × 10^−7^ S cm^−1^ (25 °C) *T_g_* = −44.8 °C; %χ_C_ = 33.6%	[115]
PEO:MC (60:40)-NH_4_I-PEG (M_w_ PEG 8000)	Solution casting	3.37 × 10^−3^ (30 °C)	*E_A_* = 0.0322 eV Unplasticized SPE: σ = 7.62 × 10^−5^ S cm^−1^ (30 °C) *E_A_* = 0.0465 eV	[116,117]
PMMA-LiClO_4_-PC [MMA]/[Li] = 4:1	Solution casting	3.52 × 10^−5^ (30 °C)	*E_A_* = 0.11 eV Conduction and relaxation via hopping mechanism	[118]
PVA-LiClO_4_-DMP (30:10:60)	Solution casting	0.15 × 10^−3^ (29 °C)	Conductivity-temperature relation obeys Vogel-Tamman-Fulcher (VTF) rule	[119]
PVA-LiCF_3_SO_3_-DMP (30:10:60)	Solution casting	~6.35 × 10^−5^ (29 °C)	Conductivity-temperature relation obeys Vogel-Tamman-Fulcher (VTF) rule	[119]
PVA-LiBF_4_-DMP (30:10:60)	Solution casting	~1.66 × 10^−5^ (29 °C)	Conductivity-temperature relation obeys Vogel-Tamman-Fulcher (VTF) rule	[119]
PEO-LiCF_3_SO_3_-15 wt.% PEG (M_w_ PEG 6000)	Ball-milling and hot press	1.71 × 10^−5^	*T_g_* = −55.8 °C; %χ_C_ = 31.6%	[38]
PEO-LiCF_3_SO_3_-20 wt.% DOP	Ball-milling and hot press	7.60 × 10^−4^	*T_g_* = −63.9 °C; %χ_C_ = 23.1%	[38]
PEO-LiCF_3_SO_3_-20 wt.% EC	Solution casting	8.12 × 10^−5^ (25 °C)	*T_g_* = −70.5 °C; %χ_C_ = 31.3% Without plasticizer: σ = 2.89 × 10^−5^ S cm^−1^ (25 °C) *T_g_* = −69.0 °C; %χ_C_ = 31.7%	[120]
Corn starch-LiPF_6_-BmImTf	Solution casting	6.00 × 10^−4^ (80 °C)	*E_A_* = 0.01 eV; *T*_g_ = −29 °C Without plasticizer: σ = ~10^−6^ S cm^−1^ (80 °C) *E*_A_ = 0.13 eV; *T_g_* = −19 °C	[121]
CMC-LiBF_4_-glycerol	Solution casting	3.70 × 10^−3^ (25 °C)	*T_g_* = 54°C *T_g_* of CMC film = 87 °C Without glycerol: σ = 8.20 × 10^−6^ S cm^−1^ (25 °C)	[122]
PVA-CH_3_COONH_4_-BmImI	Solution casting	9.63 × 10^−5^ (25 °C)	*E_A_* = 6.68 meV; %χ_C_ = 2% *D^+^* = 9.80 × 10^−12^ cm^2^ s^−1^ EDLC at scan rate 10 mV s^−1^, 200th cycle: Specific capacitance = 44.46 F g^−1^ Power density = 47.66 kW kg^−1^ Without BmImI: *σ* = 1.94 × 10^−5^ S cm^−1^ (25 °C)	[123]
Chitosan-NH_4_I-BmImI	Solution casting	3.43 × 10^−5^ (27 °C)	*E_A_* = 0.25 eV N3/TiO_2_/SPE/Pt DSSC (light intensity of 100 mW cm^−2^): OCV = 0.58 V; *J_sc_* = 3.10 mA cm^−2^; *FF* = 0.41; *η* = 0.74% Without BmImI: *σ* = 3.73 × 10^−7^ S cm^−1^ (27 °C)	[124]
PEO-LiTFSI-30 wt.% TCNE [EO]/[Li] = 12:1	Solution casting	1.11 × 10^−4^ (25 °C)	*T_g_* = −46.21 °C Without TCNE: *σ* = 1.21 × 10^−5^ S cm^−1^ (25 °C) *T_g_* = −43.03°C	[125]
Chitosan-NaI-I_2_-EMImSCN	Solution casting	2.60 × 10^−4^ (RT)	N719/TiO_2_/SPE/Pt DSSC (light intensity of 100 mW cm^−2^): OCV = 0.53 V; *J_sc_* = 2.62 mA cm^−2^; *FF* = 0.52; *η* = 0.73% Without EMImSCN: *σ* = 1.21 × 10^−5^ S cm^−1^ (25 °C) OCV = 0.35 V; *J_sc_* = 1.05 mA cm^−2^; *FF* = 0.34; *η* = 0.13%	[126]
PVDF-KI-I_2_-30 wt.% MCP	Solution casting	4.58 × 10^−5^ (30 °C)	*T_m_* = 152.8 °C; %χ_C_ = 51.49% N3/TiO_2_/SPE/Pt DSSC: *η* = 2.65% at 60 mW cm^−2^ Without MCP: *σ* = 4.79 × 10^−6^ S cm^−1^ (30 °C) *T_m_* = 170.5 °C; %χ_C_ = 63.80% *η* = 1.18% at 60 mW cm^−2^	[127]
PVDF-HFP-KI-TBAI-I_2_- 4 wt.% ATDT	Solution casting	2.82 × 10^−4^ (30 °C)	Porous surface morphology N3/TiO_2_/SPE/Pt DSSC: *η* = 4.64% at 60 mW cm^−2^ Without ATDT: σ = 9.99 × 10^−6^ S cm^−1^ (30 °C) *η* = 1.88% at 60 mW cm^−2^	[128]
PVDF-HFP-NaI-EMImSCN	Solution casting	2.65 × 10^−3^ (30 °C)	Electrochemical stability window 3.6 V EDLC at scan rate 0.05 mV s^−1^: Specific capacitance = 2.36 F g^−1^	[129]
P(GMA-co-MMA)-LiClO_4_-EC	Solution casting	3.00 × 10^−4^ (RT)	Electrochemical stability window 3.8 V Without EC: *σ* = 8.70 × 10^−6^ S cm^−1^ (RT)	[65]
PVAc:PMMA (70:30)-LiCl-EC	Solution casting	1.03 × 10^−4^ (30 °C)	*T_g_* = 26.6 °C; t_+_ = 0.98 *D_+_* = 7.00 × 10^−11^ cm^2^ s^−1^; *μ* = 2.68 × 10^−9^ cm^2^ V^−1^ s^−1^; *n* = 2.35 × 10^22^ cm^−3^ Without plasticizer: *σ* = 1.03 × 10^−5^ S cm^−1^ (30 °C) *T_g_* = 42.6 °C; t_+_ = 0.97 *D_+_* = 6.93 × 10^−11^ cm^2^ s^−1^; *μ* = 2.65 × 10^−9^ cm^2^ V^−1^ s^−1^; *n* = 2.35 × 10^22^ cm^−3^	[99]

PGMA—poly glycidyl methacrylate; SN—succinonitrile; HEC—hydroxyethylcellulose; PVB—polyvinyl butyral; NH_4_CF_3_SO_3_—ammonium trifluoromethane sulfonate; BMATFSI—butyl trimethylammonium bis(trifluoromethanesulfonyl)imide; LiBETI—lithium bisperfluoroethylsulfonylimide (Li(C_2_F_5_SO_2_)_2_N); LiCoO_2_ –lithium cobalt oxide; S_2_TFSI—triethylsulfonium bis(trifluoromethylsulfonyl)imide; LiFSI—lithium bis(fluorosulfonyl)imide; N_1222_FSI—triethylmethylammonium bis(fluorosulfonyl)imide; DMP—dimethyl phthalate; DOP—dioxy phthalate; BmImTf—1-butyl-3-methylimidazolium trifluoromethanesulfonate; CH_3_COONH_4_—ammonium acetate; BmImI—1-butyl-3-methylimidazolium iodide; TCNE—tetracyanoethylene; EMImSCN—1-ethyl 3-methylimidazolium thiocyanate; MCP—2-mercatopyridine; ATDT—5-amino-1,3,4-thiadiazole-2-thiol.

**Table 7:** Various SPEs systems with fillers added.

Electrolyte System	Preparation Method	Conductivity, *σ* (S cm^−1^)	Important Findings	Reference
PEO-LiCF_3_SO_3_-TiO_2_ [EO]/[Li] = 9:1	Solution casting	4.90 × 10^−5^ (30 °C)	*E_A_* = 0.82 eV; *T_g_* = −46 °C; *T_m_* = 60 °C	[43]
PEO-LiN(CF_3_SO_2_)_2_-SiO_2_ [EO]/[Li] = 8:1	Solution casting	1.40 × 10^−4^	*E_A_* = 0.54 eV; *T_g_* = −43 °C Without filler: *σ* = 1.50 × 10^−5^ S cm^−1^ (27 °C); *E_A_* = 0.60 eV; *T_g_* = −49 °C	[177]
PEO-LiClO_4_-SiO_2_ [EO]/[Li] = 8:1	Solution casting	2.50 × 10^−5^	*E_A_* = 0.55 eV; *T_g_* = −44 °C Without filler: *σ* = 9.70 × 10^−7^ S cm^−1^; *T_g_* = −46 °C	[177]
PEO-LiClO_4_-LLTO	Solution casting	7.99 × 10^−4^ (70 °C)	LLTO nanoparticles was synthesized via Pechini precursor method. PCTB/SPE/Li cell (60 mA g^−1^; 70 °C): Discharge capacity = 104 mAh g^−1^ 90% capacity retention after 300th cycle	[178]
PEO-LiPF_6_-EC-αCNT	Solution casting	1.30 × 10^−3^ (25 °C)	αCNT was synthesized via chemical route Bandgap energy = 4.42 eV (indirect) and 4.60 eV (direct)	[103]
Chitosan-LiClO_4_-glycerol-Ag nanowires	Solution casting	~2.07 × 10^−5^ (25 °C)	Diameter of Ag nanowires = 20 nm Elastic modulus = ~3.5 MPa	[107]
Chitosan-LiClO_4_-glycerol-Ag nanospheres	Solution casting	~1.00 × 10^−4^ (25 °C)	Diameter of Ag nanospheres = 100 nm Elastic modulus = ~1.0 MPa	[107]
PVA: PVP (1:1)-MgCl_2_-CuS	Solution casting	3.32 × 10^−7^ (RT) 1.85 × 10^−3^ (protonic σ at RT)	CuS nanoparticles (particle size 20 nm) was synthesized via microwave irradiation method More amorphous after CuS addition Suitable for solid state magnesium batteries, supercapacitors and PEMFC	[179]
PEO-PMMA (1:1)-LiClO_4_-3 wt.% SiO_2_ [EO + C=O]/[Li] = 9:1	Solution casting + melt press	0.51 × 10^−5^ (27 °C)	SiO_2_ particle size < 15 nm *E_A_* = 0.32 eV; *t_Li+_* = 0.9894 Electrochemical stability window 6.18 V Without filler: *σ* = 1.72 × 10^−5^ S cm^−1^ (27 °C); *E_A_* = 0.37 eV	[180,181]
PEO-PMMA (1:1)-LiClO_4_-3 wt.% Al_2_O_3_ [EO + C=O]/[Li] = 9:1	Solution casting + melt press	0.38 × 10^−5^ (27 °C)	Al_2_O_3_ particle size < 50 nm *E_A_* = 0.30 eV; *t_Li+_* = 0.9836 Electrochemical stability window 6.00 V	[180,181]
PEO-PMMA (1:1)-LiClO_4_-3 wt.% ZnO [EO + C=O]/[Li] = 9:1	Solution casting + melt press	1.67 × 10^−5^ (27 °C)	ZnO particle size < 100 nm *E_A_* = 0.27 eV; *t_Li+_* = 0.9857 Electrochemical stability window 6.06 V	[180,181]
PEO-PMMA (1:1)-LiClO_4_-3 wt.% SnO_2_ [EO + C=O]/[Li] = 9:1	Solution casting + melt press	1.00 × 10^−5^ (27 °C)	SnO_2_ particle size = 100 nm *E_A_* = 0.38 eV; *t_Li+_* = 0.9881 Electrochemical stability window 6.10 V	[180,181]
PEO-PMMA (1:1)-LiClO_4_-10 wt.% MMT [EO + C=O]/[Li] = 12:1	Microwave irradiation + Solution casting and melt press	3.79 × 10^−6^ (27 °C)	MMT is layered nanosheet which is compatible with PEO polymer chain Without filler: *σ* = 1.99 × 10^−6^ S cm^−1^ (27 °C)	[100]
PVDF-CA-LiTFSI-OMMT		3.40 × 10^−4^ (25 °C)	*E_A_* = 0.45 eV; *t_Li+_* = 0.315 Tensile strength = 44.89 MPa Electrochemical stability window 4.20 V LiFePO_4_/SPE/Li: Discharge capacity = 112.9 mAh g^−1^ (0.5 C; 100th cycle) Without filler: *E_A_* = 0.53 eV; *t_Li+_* = 0.120 Tensile strength = 34.93 MPa	[182]
PEO-PMMA (1:1)-LiBF_4_-EC-MMT PEO-PMMA (1:1)-LiBF_4_-EC-MMT	Solution cast method ultrasonic-microwave irradiation solution cast method	9.17 × 10^−6^ (27 °C) 6.43 × 10^−6^ (27 °C)	Ion-dipolar complexes intercalated in MMT network in SPE prepared via solution cast method. Exfoliated MMT structure in SPE by ultrasonic-microwave irradiation solution cast method	[183]
PEO-LiCF_3_SO_3_-20 wt.% Al_2_O_3_	Ball-milling and hot press	8.64 × 10^−5^ (RT)	*T_g_* = −65.3 °C; %χ_C_ = 18.6%	[38]
PEO-LiCF_3_SO_3_-EC-15 wt.% Al_2_O_3_	Solution casting	5.07 × 10^−4^ (25 °C)	*T_g_* = −72.0 °C; %χ_C_ = 31.1% Without filler: *σ* = 8.12 × 10^−5^ S cm^−1^ (25 °C) *T_g_* = −70.5 °C; %χ_C_ = 31.3%	[120]
PMA:PEG (65:35)-NaClO_4_-nano α-Al_2_O_3_	Solution casting	1.76 × 10^−4^ (70 °C)	*E_A_* = 0.38 eV Tensile strength = 50.79 MPa Electrochemical stability window 4.50 V Na_3_V_2_(PO_4_)_3_/SPE/Na cell (0.5 C, 70 °C): Discharge capacity = 85 mAh g^−1^ 94.1% capacity retention after 350th cycle Without filler: *σ* around 10^−5^ S cm^−1^ (70 °C)	[184]
PEO-PVP-NaIO_4_-GO	Solution casting	1.00 × 10^−6^ (30 °C)	Without filler: *σ* = 1.57 × 10^−7^ S cm^−1^ (30 °C)	[185]
PEO-LiTFSI-*g*-C_3_N_4_ [EO]/[Li] = 20:1	Solution casting	3.06 × 10^−5^ (25 °C) 2.50 × 10^−4^ (60 °C)	g-C_3_N_4_ was synthesized via thermal polycondensation route *t_Li+_* = 0.69; *T_g_* = −41.1 °C Tensile strength = 3.97 MPa Electrochemical stability window 5.12 V at 60 °C FeF_3_/SPE/Li cell (1 C, 60 °C): Capacity 300 mAh g^−1^ after 200th cycle Without g-C_3_N_4_: *σ* = 2.32 × 10^−6^ S cm^−1^ (25 °C) *t_Li+_* = 0.25; *T_g_* = −40.7 °C Tensile strength = 0.92 MPa Electrochemical stability window 4.60 V at 60 °C	[186]
PEO-LiTFSI-Li_10_GeP_2_S_12_ [EO]/[Li] = 18:1	Solution casting	1.18 × 10^−5^ (25 °C) 1.21 × 10^−3^ (80 °C)	Li_10_GeP_2_S_12_ (particle size 2–3 μm) was synthesized via solid state reaction method *T_g_* = −41.6 °C; %χ_C_ = 42.14% *t_Li+_* = 0.26 (80 °C) Electrochemical stability window 5.70 V LiFePO_4_/SPE/Li cell (0.5 C, 60 °C): Discharge capacity = 137.4 mAh g^−1^ 92.5% capacity retention after 50th cycle Without filler: *σ* = 6.16 × 10^−6^ S cm^−1^ (25 °C); = 7.98 × 10^−4^ S cm^−1^ (80 °C); *T_g_* = −39.6 °C; %χ_C_ = 67.07% *t_Li+_* = 0.22 (80 °C) Electrochemical stability window 4.80 V Discharge capacity = ~50 mAh g^−1^ 50.4% capacity retention after 50th cycle	[187]
PVAc:PMMA (70:30)-LiCl-TiO_2_	Solution casting	4.45 × 10^−4^ (30 °C)	*T_g_* = 28.1 °C; *t_+_* = 0.99 *D_+_* = 7.07 × 10^−11^ cm^2^ s^−1^; *μ* = 2.71 × 10^−9^ cm^2^ V^−1^ s^−1^; *n* = 2.35 × 10^22^ cm^−3^ Electrochemical stability window 2.69 V Without filler: *σ* = 1.03 × 10^−5^ S cm^−1^ (30 °C) *T_g_* = 42.6 °C; *t_+_* = 0.97 *D_+_* = 6.93 × 10^−11^ cm^2^ s^−1^; *μ* = 2.65 × 10^−9^ cm^2^ V^−1^ s^−1^; *n* = 2.35 × 10^22^ cm^−3^ Electrochemical stability window 1.69 V	[99]
PEO-LiTFSI-Ni_3_-(BTC)_2_	Hot pressing	1.40 × 10^-1^ (30 °C)	LiFePO_4_/SPE/Li cell (1 C): Discharge capacity = 75 mAh g^-1^	[188]

LLTO—Li_0.3_La_0.566_TiO_3_; PCTB—poly(2-chloro-3,5,6-trisulfide-1,4-benzoquinone); αCNT—amorphous carbon nanotubes; CuS—copper sulfate; CA—cellulose acetate; OMMT—organic modified montmorillonite; MMT—montmorillonite; Al_2_O_3_—nano alumina; NaIO_4_—sodium periodate; GO—graphene oxide; PMA—poly(methacrylate); *g*-C_3_N_4_—polymeric graphitic carbon nitride; Ni_3_-(BTC)_2_—nickel-1,3,5-benzene trcarboxxylate metal organic framework.

## Data Availability

Not applicable.
